# Genome-wide landscape of position effects on heterogeneous gene expression in *Saccharomyces cerevisiae*

**DOI:** 10.1186/s13068-017-0872-3

**Published:** 2017-07-18

**Authors:** Xiao-Le Wu, Bing-Zhi Li, Wen-Zheng Zhang, Kai Song, Hao Qi, Jun-biao Dai, Ying-Jin Yuan

**Affiliations:** 1 Key Laboratory of Systems Bioengineering (Ministry of Education), School of Chemical Engineering and Technology, Tianjin University, Tianjin, 300072 People’s Republic of China; 2 SynBio Research Platform, Collaborative Innovation Center of Chemical Science and Engineering (Tianjin), Tianjin University, Tianjin, 300072 People’s Republic of China; 3 Key Laboratory of Industrial Biocatalysis (Ministry of Education) and Center for Synthetic and Systems Biology, School of Life Sciences, Tsinghua University, Beijing, 100084 People’s Republic of China

**Keywords:** Position effect, Genome, Gene expression, Synthetic biology, Yeast

## Abstract

**Background:**

Integration of heterogeneous genes is widely applied in synthetic biology and metabolic engineering. However, knowledge about the effect of integrative position on gene expression remains limited.

**Results:**

We established a genome-wide landscape of position effect on gene expression in *Saccharomyces cerevisiae*. The expression cassette of red fluorescence protein (RFP) gene was constructed and inserted at 1044 loci, which were scattered uniformly in the yeast genome. Due to the different integrative loci on the genome, the maximum relative intensity of RFP is more than 13-fold over the minimum. Plots of the number of strains to RFP relative intensity showed normal distribution, indicating significant position effect on gene expression in yeast. Furthermore, changing the promoters or reporter genes, as well as carbon sources, revealed little consequences on reporter gene expression, indicating chromosomal location is the major determinant of reporter gene expression.

**Conclusions:**

We have examined the position effects to integration genes expression in large number loci around whole genome in *S. cerevisiae*. The results could guide the design of integration loci for exogenous genes and pathways to maximize their expression in metabolic engineering.

**Electronic supplementary material:**

The online version of this article (doi:10.1186/s13068-017-0872-3) contains supplementary material, which is available to authorized users.

## Background

Are genes scattered within the genome randomly? The answer is probably no. However, the pattern and underling principle of gene distribution remain largely elusive. The genomic locations of specific sequences are important for their regulatory functions [1], and it is reasonable that the genomic location of a coding sequence plays important roles on its expression. DNA sequences, such as promoters, enhancers, insulators and so on [2–4], are usually used as the common regulatory factors to control gene expression. The effect of genomic location will open another door to regulate gene expression.

For decades, regulation of the heterogeneous or homogenous gene expression is the key approach for biologists and bioengineers [5, 6]. In consideration of stability and efficiency of gene expression, chromosomal integration is preferred to episomal plasmid [7–9]. The structure of chromosome affects and regulates the cellular processes, including gene expression [10–12], DNA replication [13, 14] and transformation efficiency [15, 16]. The position effect refers to that the heterologous genes inserted at different loci of chromosomes presented various expression levels, which have been reported in *Escherichia coli* [17, 18], S*almonella typhimurium* [19, 20], *Bacillus subtilis* [21], *Lactobacillus lactis* [22], *Saccharomyces cerevisiae* [22–26], *Drosophila melanogaster* [27], mouse cells [28], and human cells [29].

In *S. cerevisiae*, the position effects on gene expression have drawn attention for decades due to its wide application. Flagfeldt et al. characterized 20 different integration sites using *LacZ* expression variation [24]. Thompson and Gasson analyzed 18 loci and found a 14-fold expression variation due to different integrative loci [22]. The position effects on gene expression were also investigated in one chromosome [23, 25]. However, there are nearly 6000 genes distributed across 16 chromosomes in *S. cerevisiae* genome. A systematic analysis the genome-wide landscape of position effect in *S. cerevisiae* is required. Chen used GFP to characterize the position effects at 482 different loci in the yeast genomic scale, approximately one third of which were on the chromosome (CHR) I and VI [26].

In this study, we characterized the position effects of 1044 uniformly scattered loci with red fluorescence protein (RFP) as the reporter gene. It reveals the genome-scale landscape of position effect on gene expression, and provides new reference to control gene expression during genetic engineering in *S. cerevisiae*.

## Results and discussion

### Expression of RFP at different chromosome loci

**Fig. 1:**
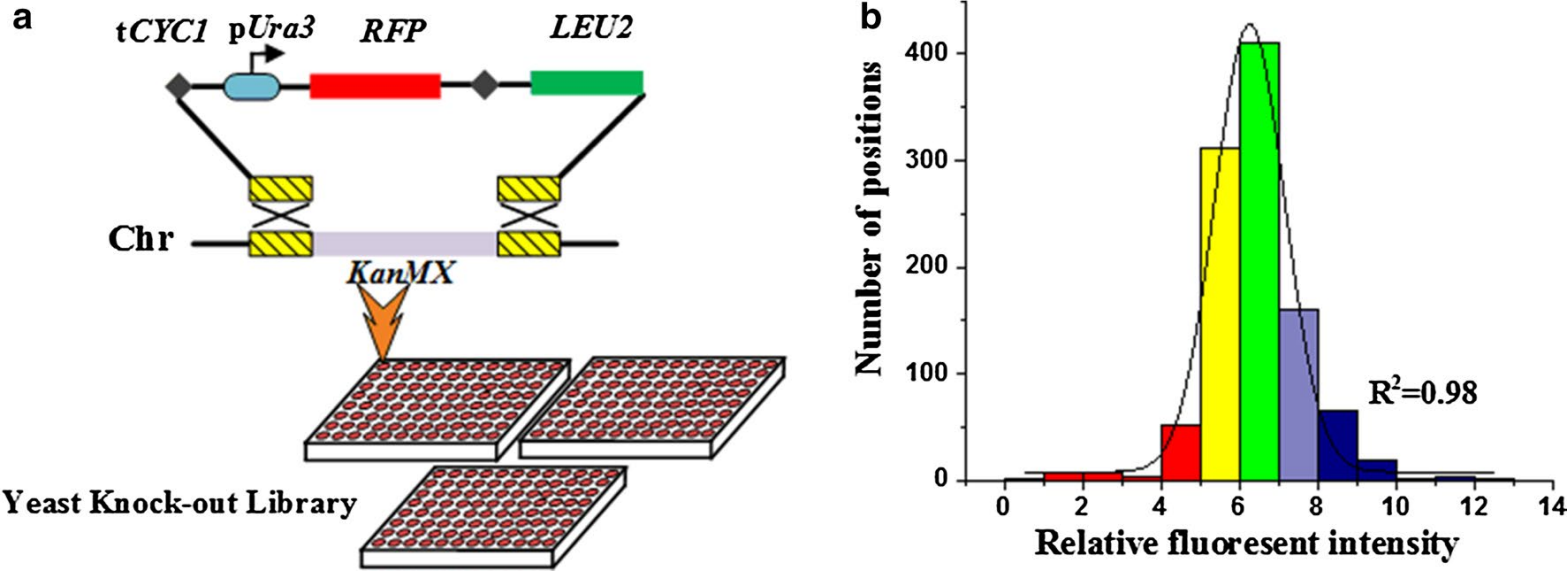
Basic configuration of vectors and homologous recombination approach. **a** Targeted integration of *RFP* reporter gene cassette. **b** The numbers of positions with different relative fluorescence intensities. *Column color* indicates relative fluorescence intensity. *Red*, *yellow*, *green*, *violet* and *blue* represent extreme low (0–5), low (5–6), moderate (6–7), high (7–8), extreme high (8–13)

In this study, *RFP* gene with URA3 promoter was integrated into deletion strains from the Yeast Knock-Out library by replacing the *kanMX* gene (Fig. 1a). To avoid the interference from the native promoter, CYC1 terminator was inserted upstream of the URA3 promoter to prevent the potential interruption from the native promoters or the read-through of the native terminators (Additional file 1: Figure S1). RFP, instead of green fluorescent protein (GFP), was used as the reporter, because GFP measurement is prone to be interrupted by auto-fluorescence of *S. cerevisiae* (Additional file 1: Figure S2). Compared with previous studies on position effects [24], variation of position effects on reporter gene expression was observed in this study.

To investigate the genomic landscape of position effects, 1044 loci were selected among 16 chromosomes and the interspace between two loci is about 12 kb. A list of selected locus was collected in the Additional file 1: Table S1. The relative fluorescence intensity was used to represent the expression level of each integrant (Additional file 1: Table S2).

The strains with the reporter gene inserted in different locus exhibited distinct relative fluorescence intensities, ranging from 0.98 to 12.98 and divided into five groups according to the relative fluorescence intensity, including extreme high (blue), high (violet), moderate (green), low (yellow) and extreme low (red), as shown in Fig. 1b. The number of strains in each group was 91, 161, 410, 311 and 71, and the corresponding proportions were 8.7, 15.4, 39.3, 29.8 and 6.8%, respectively. The majority strains were allocated into the high, moderate and low groups, accounting for 84.5% of the entire library. However, the remaining two groups (extreme high and extreme low) caught our attention although they accounted for only 15.5%.

**Fig. 2:**
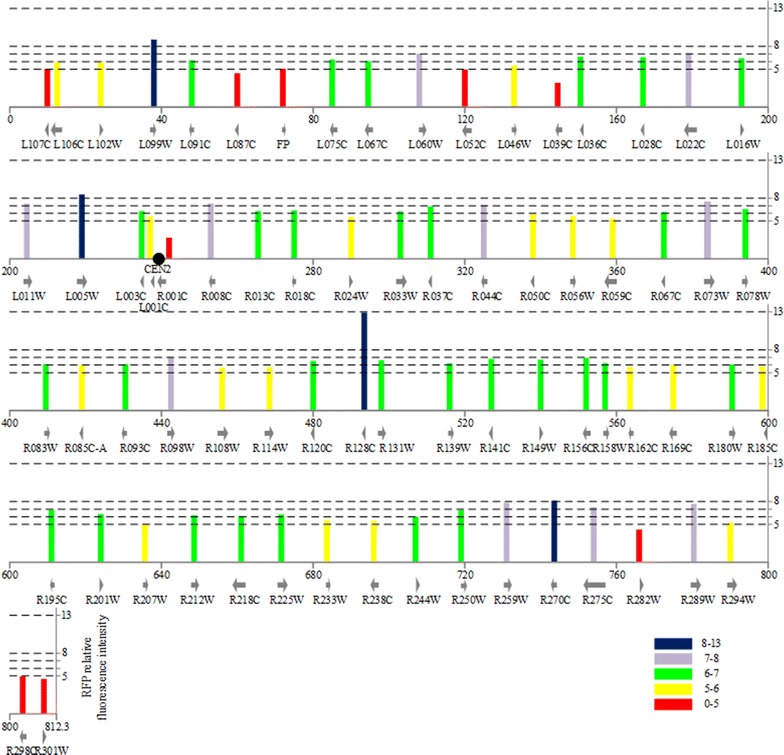
Region specificity on expression of p*URA3*-*RFP* cassette at various positions on CHR II. Tag height is degree of relative fluorescence intensity. *Red*, *yellow*, *green*, *violet* and *blue* indicated extreme low (0–5), low (5–6), moderate (6–7), high (7–8), extreme high (8–13), respectively. The locations of ORFs on chromosome II and their directions of transcription are presented with *arrows*. Centromere is located on the chromosome II by a *solid dot*

**Fig. 3:**
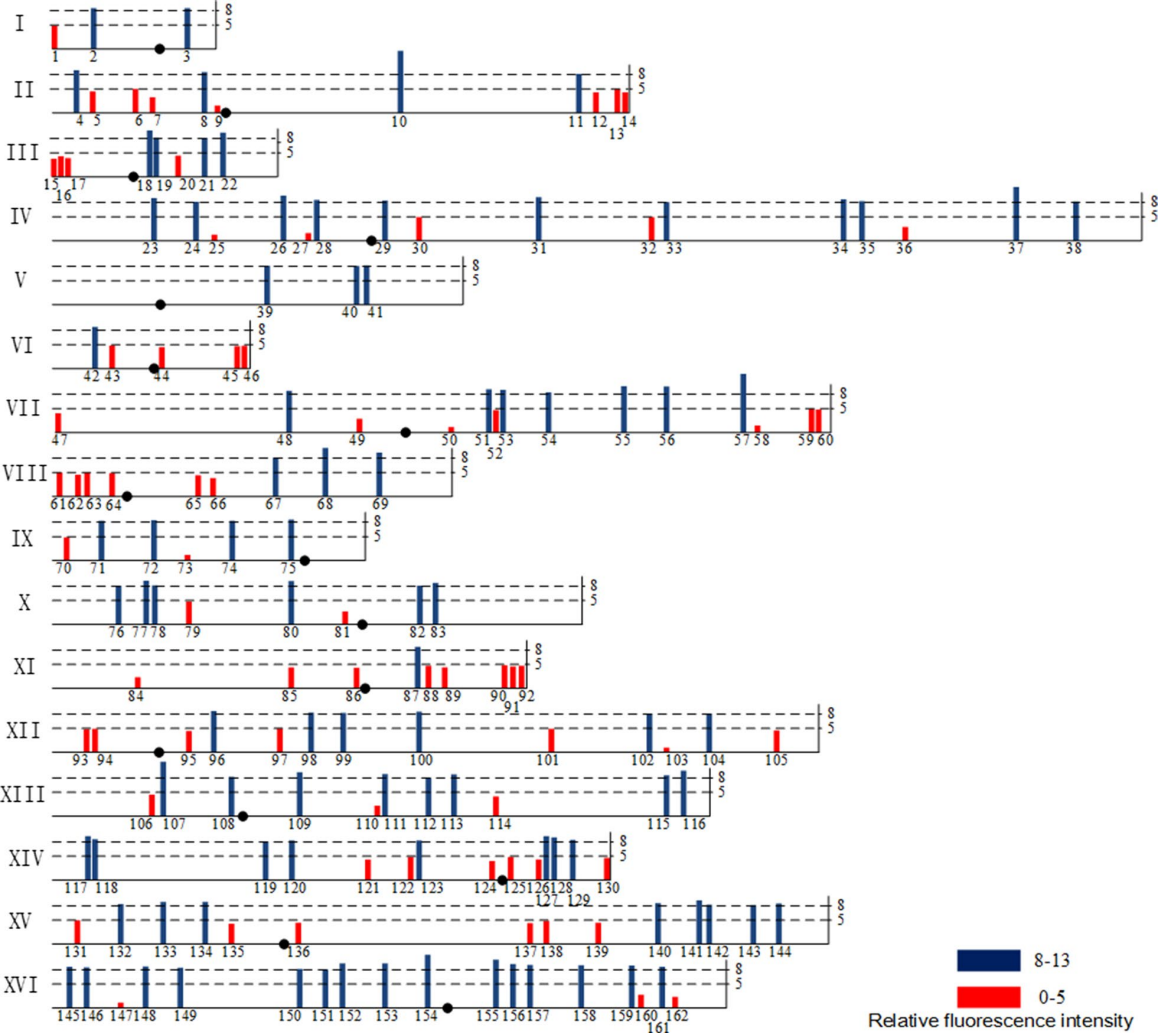
Region specificity on expression of p*URA3*-*RFP* cassette at various positions on genome of *S. cerevisiae*. Tag height is degree of relative fluorescence intensity. *Red* and *blue* indicated extreme low (0–5) and extreme high (8–13), respectively. Centromere is located on the chromosome by a *solid dot*. (The detail positions are in the Additional file 1: Table S4)

We compared the relative fluorescence intensity of strains with different integrative locus in 16 chromosomes. The RFP relative fluorescence signals of 71 loci within CHR II were shown in Fig. 2. RFP expression at different positions varied significantly and the distribution of RFP expression was roughly random except for the regions near the telomere and centromere. Similar results were also observed in other 15 chromosomes (Fig. 3). To further confirm this finding, we calculated the percentage of strains in the five groups for loci within the region of 20 kb flanking centromeres and 10 kb away from telomeres. Table 1 indicated that these loci are over-represented by lower relative fluorescence intensity (51.3%), while only 29.8% loci in the whole genome were at this level of intensity. Furthermore, no locus near the telomere and centromere exhibited extreme high relative fluorescence intensity (Table 1). The result of the Chi-square consistency test was 51.26 which is much bigger than the corresponding threshold value (9.49, when *α* = 0.05, *α* is the significance level). Consequently, the null hypothesis was denied, which meant that the distribution of the specific loci was independent from the distribution of the whole genome loci.

**Table 1:** Distributions of fluorescence intensities within the loci over all the whole genome and the loci near telomere and centromere (TEL/CEN)

Groups	Genome (%)	TEL/CEN
Extreme high	8.7	0
High	15.4	6.0%
Moderate	39.3	25.6%
Low	29.8	51.3%
Extreme low	6.8	17.1%

### Loci with extreme high or extreme low gene expression levels

A total of 162 loci fell in extreme high or extreme low groups and were marked on the 16 chromosomes in Fig. 3. The locus with the highest expression level was YBR128C on CHR II with the relative fluorescence intensity of 12.98 and the loci exhibiting high expression level included YDR448W, YGR240C, YHR142W, YML059C, YPL014W, and YPR028W, which were distributed among the 16 chromosomes.

The loci with low gene expression were mostly restricted to the regions near the telomeres and centromeres. On the CHR XI, there are eight loci in the extreme low group but only one locus in the extreme high group. Moreover, the extreme low locus was lacking on CHR V.

It was reported that the genome 3D structure affected gene expressions [30]. Among the loci identified here, 16 were overlapped with the top 88 loci with the highest interaction frequencies. We found that all 16 loci were in the group of extreme low, low or moderate expression levels. The results suggested that the chromosome interactions in 3D chromosome conformation would affect position effect on gene expressions, and higher interaction frequencies could possibly reduce gene expression.

It is known that telomeres and centromeres could inhibit the expression of genes nearby [26], which is consistent with our finding that the reporters integrated near these regions have lower expression. On the other hand, it has been reported that the high gene expressions were related with the location near to autonomously replicating sequence (ARS) [24]. However, we found not all the reporter genes around ARS exhibited high level of expression, especially the ARS near the telomere and centromere. The influence discrepancy could due to the different effectiveness of various ARS in *S. cerevisiae*, as reported [31–33]. We further analyzed all the strains with the reporter gene at the loci near the high effective ARS in CHR III and CHR VI according to previous studies [31–33], and no strains fell into the group of the extreme low (Table 2).

**Table 2:** The relative fluorescence intensities of the reporter gene integrated at the loci near the ARS on CHR III and CHR VI

ARS	Loci	Distance (bp)	Relative fluorescence intensity	Groups
ARS305	YCL051W	1892	6.42 ± 0.16	Moderate
	YCL044C	7516	6.54 ± 0.26	Moderate
ARS306	YCL027W	1117	7.38 ± 0.42	High
	YCL025C	1341	5.99 ± 0.16	Low
ARS307	YCL016C	12,964	7.05 ± 0.37	High
	YCL005W	Partial overlap	5.74 ± 0.47	Low
ARS309	YCR004C	11,725	6.41 ± 0.22	Moderate
	YCR010C	153	6.82 ± 0.51	Moderate
ARS310	YCR024C-A	3436	5.01 ± 0.29	Low
	YCR027C	22	5.58 ± 0.26	Low
ARS315	YCR051W	10,077	8.14 ± 0.12	Extreme high
	YCR061W	502	5.08 ± 0.32	Low
ARS319	YCR107W	1917	5.07 ± 0.59	Low
ARS603	YFL036W	5922	8.70 ± 0.34	Extreme high
	YFL033C	254	6.87 ± 0.06	Moderate
ARS606	YFR006W	9795	5.27 ± 0.36	Low
	YFR012W	87	5.57 ± 0.32	Low
ARS607	YFR021W	3081	6.58 ± 0.45	Moderate
	YFR025C	4238	5.77 ± 0.23	Low

### Robustness of the position effect

**Fig. 4:**
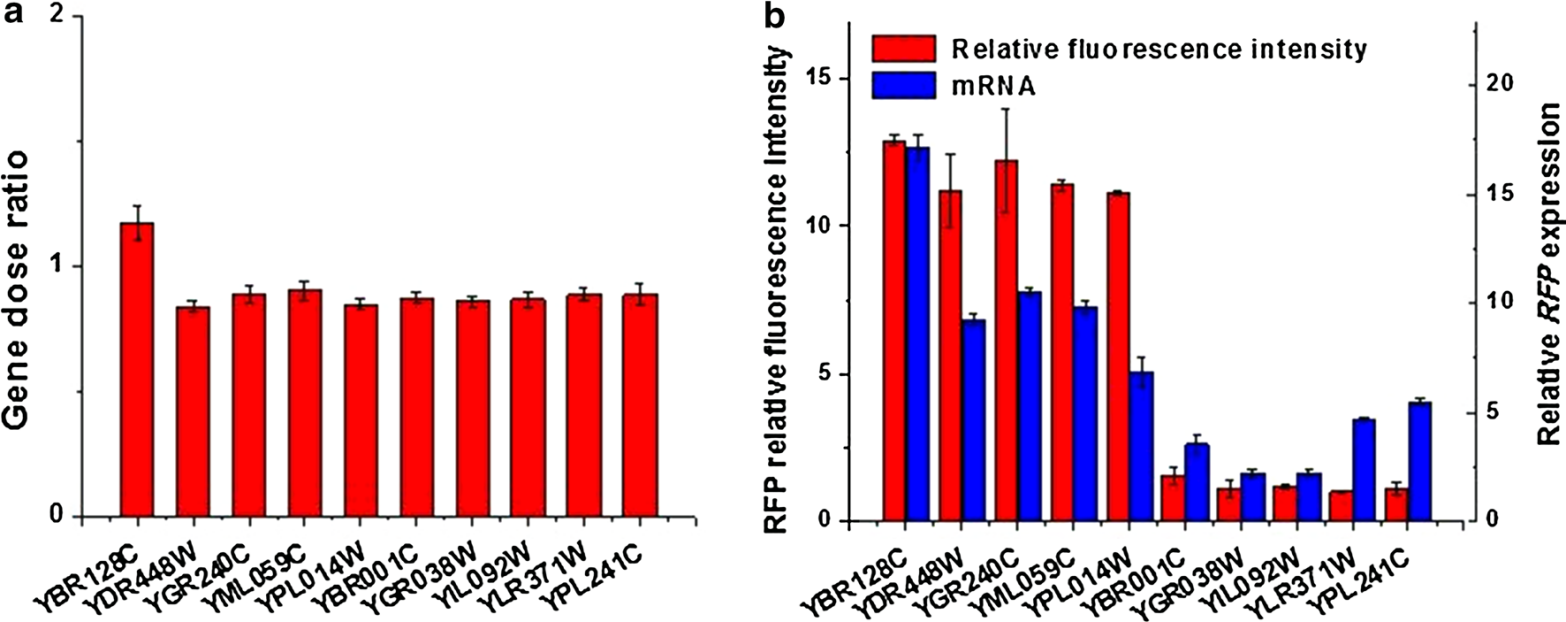
Position effect occur at the level of transcription. **a** Copy numbers of the *RFP* gene at ten loci. **b** Relative fluorescence intensity and *RFP* mRNA expression at ten loci

Gene expressions are affected by many factors, such as the gene copy number and the strength of promoter. To eliminate the noise effects from copy number on gene expression, we examined how many copies of reporter genes were inserted into the genome in ten selected strains, which included five strains with extreme high relative fluorescence intensity (YBR128C, YDR448W, YGR240C, YML059C and YPL014W) and five strains with extreme low relative fluorescence intensity (YBR001C, YGR038W, YIL092W, YLR371W and YPL241C). As Fig. 4a shows, the copy number of the reporter gene was all around 0.9. The corresponding standard deviation value was only 0.097 (<10% of the mean value), which meant the dispersion of these values was very small. Therefore, the number of reporter genes was not the reason for the different relative fluorescence intensities in these strains. The relative fluorescence intensity was mainly due to the position effects.

The relative fluorescence was used to evaluate the relative position effect on gene expression here. We next examined the amount of RNA transcripts of the reporter gene in ten strains. Results indicated that the five strains with extreme high relative fluorescence intensity also accumulated significantly more RNAs than the five strains with extreme low relative fluorescence intensity (Fig. 4b), despite that the difference was much smaller than that of relative fluorescence. The Pearson’s correlation coefficient between the relative fluorescence intensity and the corresponding RNA transcripts was as high as 0.85. When the Pearson’s correlation coefficient is bigger than 0.5, it indicates a very strong positive linear relationship between these two sets of values.

**Fig. 5:**
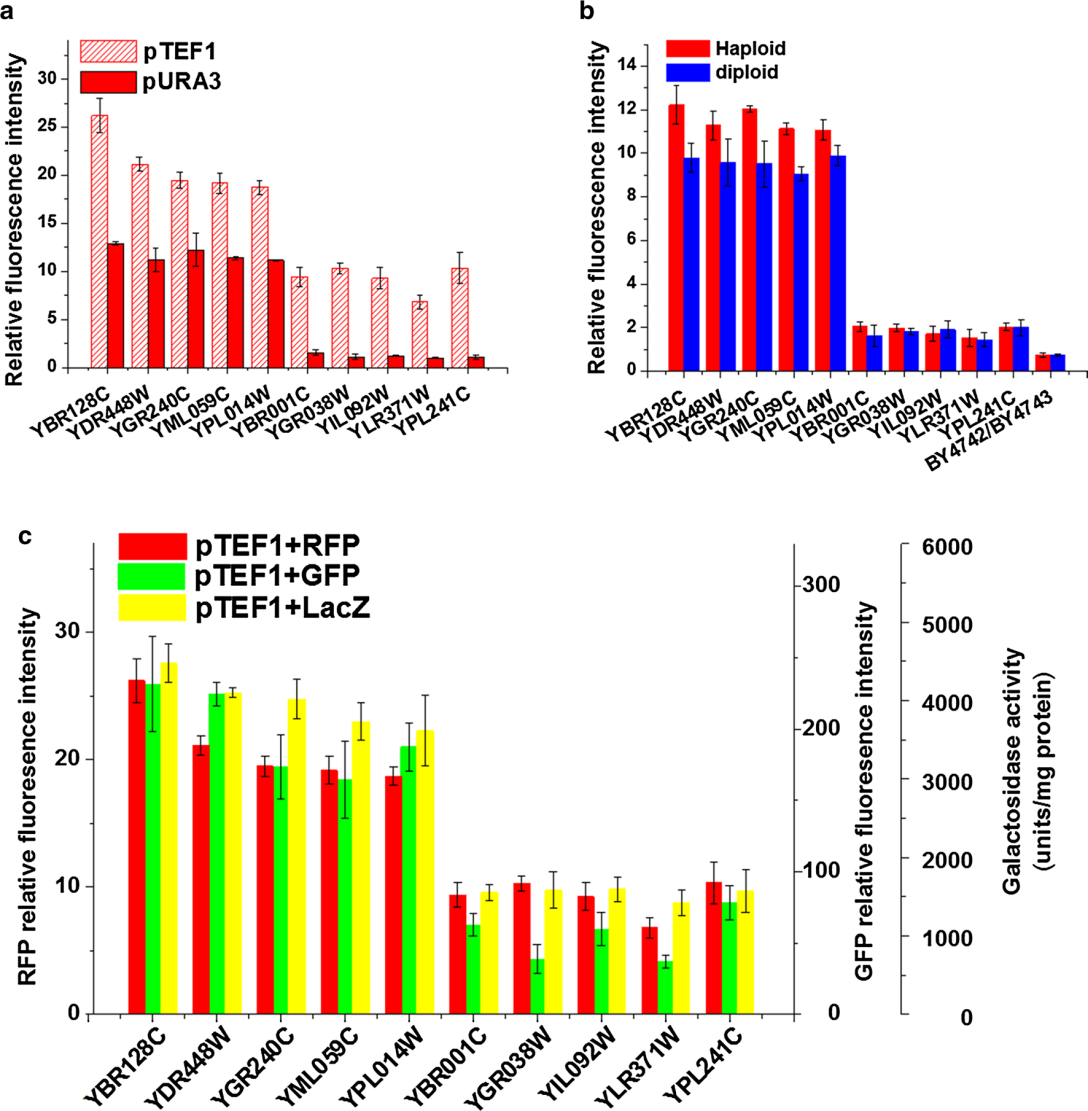
The robustness of the position effect. **a** The influence of promoter on position effect. **b** The influence of gene deletion on position effect. **c** The influence of report genes on position effect

To evaluate the possibility that the position effects might be influenced by the expression level of the reporter gene, an alternative promoter pTEF1, was used to control the reporter gene instead of pURA3. As shown in Fig. 5a, although the variation of intensities among strains carrying pTEF1 was reduced, the pattern of relative fluorescence intensities in these strains was similar to those with pURA3. The biggest difference on relative fluorescence intensity among the ten strains with pURA3 was 13.2-fold, whereas it was 3.9-fold among the strains with pTEF1. This result implied that with the increasing expression level of the reporter, the position effect still exists but its contribution was largely diminished in *S. cerevisiae*. Furthermore, we used Crisper-cas9 system in removing the marker gene (*LEU2*) which was neighbored the report gene. The result implied that the position effect was still robust in extreme high and extreme low group (Additional file 1: Figure S3).

In addition, besides RFP, two other reporter genes, *GFP* and *LacZ*, were applied to investigate the influence of different reporter genes on position effect. As shown in Fig. 5c, the resulting pattern of position effect was similar among all three types of reporters, which indicated that position effect was not a consequence from a particular reporter.

The report genes were integrated into the knock-out yeast strains with genes deleted at different locus, which might cause the possibility that the position effects were partly resulted from the gene deletion. Ten heterozygote diploids were applied to determine the potential effects of the deleted genes. As shown in Fig. 5b, similar gene expression levels were observed between the haploid and diploid strains. Therefore, the reporter gene expression was not significantly affected by the gene deletion.

The Kullback–Leibler divergence values of the data in the three subplots in Fig. 5 were 0.07, 0.00 and 0.01, which meant that there was no information gained or lost using any set of samples. For example, in Fig. 5a, there was no information gained or lost using TEF1 or URA3 as promoters. In other words, the promoters had no effect on the relative fluorescence intensity here. Neither did the locus of deleted genes nor the reporter genes.

Furthermore, it is known that different carbon sources were often used for the biosynthesis of different chemicals in yeast, which requires the cells to shift the metabolic pathways to utilize these carbon sources. To investigate whether the position effect could also be affected by carbon sources, we characterized the *RFP* gene expression integrated into CHR II under different carbon sources. We found that the ratio of *RFP* expression between glucose and glycerin fell within a narrow range 0.6–1.7, indicating that position effect is almost independent on carbon source (Additional file 1: Table S4).

## Conclusions

*Saccharomyces cerevisiae* is also a model system for basic researches in life sciences. Different relative fluorescence intensities were observed depending on the integration sites, revealing the genomic landscape of position effects. The observations of position effect provide additional regulatory mechanism of gene expression. Besides the known regions such as telomere and centromere which could silence the adjacent genes, many other loci were identified. The mechanism how genes inserted within these loci are repressed remains unknown and further studies will be required. We also confirmed the robustness of the position effects by testing different promoters, reporter genes, and different carbon sources.

## Methods

### Strains and media

All the strains with reporter genes were developed by homologous integration of the reporter genes (RFP) at different locus of *kanMX* cassettes in strains from Yeast Knock-Out (YKO) collection. The strains of YKO were grown in YPD (glucose 20 g/L, yeast extract 10 g/L, tryptone 20 g/L) with 200 μg/mL G418. The strains with reporter genes were grown in synthetic dextrose (SD) medium containing 6.7 g/L yeast nitrogen base without amino acids, 2 g/L complete supplement mixture (without histidine, leucine, tryptophan, uracil), 20 g/L glucose, 20 mg/L histidine, 20 mg/L tryptophan, and 20 mg/L uracil.

### Construction of expression cassettes and reporter gene integration

The plasmid carrying the reporter gene was constructed on the basis of the plasmid pUC19, which included two homologous arms, kanMX-L and kanMX-R, reporter genes (*RFP*, *GFP* or *LacZ*) with promoters (p*URA3* or p*TEF1*), and a screening gene *LEU2* (Fig. 1a). The enzymatic sites *sac*I and *BamH*I were inserted to the ends of the cassette, respectively. The plasmid was digested by *sac*I and *BamH*I, and the DNA fragments with reporter genes were purified and used to the following transformation procedure.

### Transformation protocol

The transformations were performed individually in 96-well plates [15, 34]. The 1044 selected strains from YKO were grown overnight in 150 μL of YPD with 200 μg/mL of G418 each deep. Ten microliters of strain suspension from each strain were inoculated in 150 μL of YPD with 200 μg/mL of G418 in the plate-shaking incubator for 5 h. Cells were harvested by centrifugation at 4000*g* for 2 min and washed once with sterile water. Next, cells were re-suspended in 0.1 M LiAc (lithium acetate). Cells were harvested by centrifugation at 4000*g* for 2 min and were re-suspended in 40 μL 0.1 M LiAc. The transformation mix was added to each transformation reaction (each well): 124 μL 50% PEG, 40 μL 1 M LiAc, 10 μL boiled ss-carrier DNA (10 mg/mL) and 10 μL DNA fragment. The plate was incubated at 30 °C for 30 min, and 10 μL of DMSO (dimethyl sulfoxide) was add in each transformation reaction, then the plate was heat shock at 42 °C for 30 min. Cells were harvested by centrifugation at 4000*g* for 5 min and washed once with 5 mM CaCl_2_ and water. After the transformation, cells were plated on SD-Leu plates, which were incubated for 2 days at 30 °C. The counter-selection and PCR were both used to confirm the correct insertion of reporter genes in chromosomes (PCR primers are in the Additional file 1: Table S5).

### Fluorescence assays

The fluorescence assays were performed individually in 96-well plates which were black and optically clear. The selected strains with reporter gene were grown overnight in 150 μL SD-Leu each deep. 10 μL bacterial suspension from each strain were inoculated in 150 μL SD-Leu and incubated 5 h in the plate-shaking incubator. Cells were harvested by centrifugation at 4000*g* for 2 min and re-suspended in PBS (phosphate-buffered saline). RFP fluorescence at excitation wavelength 587 nm and emission wavelength 610 nm was measured by plate reader (SpectraMax M2, molecular devices) [35, 36]. The excitation wavelength and emission wavelength of GFP fluorescence are 485 and 510 nm [37–39]. The relative fluorescence intensity was derived as fluorescence/OD_600_.

### Q-PCR

Real-time PCR was carried out on a CFX96 Cycler real-time PCR detection system (Bio-Rad Laboratories, Inc., Hercules, CA, USA), in white-walled PCR plates (96 wells). A ready to use master-mix containing a fast proof-reading polymerase, dNTPs, stabilizers, MgCl2 and SYBR^®^ Green dye was used according to the manufacturer’s instructions (Bio-Rad). Reactions were prepared in a total volume of 18 μL containing 400 nM each primer (MWG), 2 × SsoAdvanced™ SYBR^®^ Green Supermix (Bio-Rad) and 2 μL cDNA. The cycle conditions were set as follows: initial template denaturation at 95 °C for 3 min, followed by 40 cycles of denaturation at 95 °C for 10 s, and combined primer annealing/elongation at 57 °C for 20 s. The amount of fluorescence for each sample, given by the incorporation of SYBR^®^ Green into dsDNA, was measured at the end of each cycle and analyzed via CFX Manager™ software 2.1 (Bio-Rad Laboratories, Inc.). The gene copies were represented by the expression level of *RFP* gene divided by that of *ALG9* gene which performed as the internal reference gene [40].

### β-galactosidase activity assay

The β-galactosidase activity assay was performed individually in 96-well plates and used the yeast β-galactosidase assay kit (Thermo). The β-galactosidase activities were normalized to the cell density and measured in β-galactosidase activity according to the following equation: β-galactosidase activity = 1000 × *A*_420_/(*t* × *V* × OD_600_). Where *t* represents the incubation time in minutes and *V* represents the culture volume (mL) used in the assay [41, 42].

### Mating

The reporter strains were constructed by replacing the *kanMX* of Yeast Knock-Out library (BY4742) with report gene (RFP). To exclude the gene deletion effect, we test the relative fluorescence intensity of diploid strains generated by mating the reporter strain with the wild-type strain (BY4741), in which the corresponding gene was existed [43].

### Kullback–Leibler divergence

For discrete probability distributions *P* and *Q*, in the context of machine learning, the Kullback–Leibler divergence *D*_KL_(*P*||*Q*) is often called the information gain achieved if *P* is used instead of *Q* [44, 45]. The smallest limit of it is 0. The smaller its value is, the smaller the gain or loss can get using *Q* instead of *P*. If we consider the percentage of a sample’s value to the sum of the values of all samples in the same set as the contribution of that sample, we can use the Kullback–Leibler divergence to evaluate the information gain or loss between two sample sets. Because the Kullback–Leibler divergence is not symmetric, that is, *D*_KL_(*P*||*Q*) ≠ *D*_KL_(*Q*||*P*), we used *D*_KL_ = (*D*_KL_(*P*||*Q*) +*D*_KL_(*Q*||*P*))/2.

## Additional file

**Additional file 1: Figure S1.** Influence of the CYC1 terminator. **Figure S2.** Relative fluorescence intensities of the GFP and RFP at the same locus. **Figure S3.** The influence of marker gene on position effect. **Table S1.** Number of integrative loci of each chromosome. **Table S2.** The relative fluorescence intensity of 1044 loci. **Table S3.** Fit curve of the distribution of the numbers of positions with different relative fluorescence intensities. **Table S4.** The position effect of the loci with extreme low and extreme high expression. **Table S5.** The effect of different carbon sources on position effect of the loci in CHR II. **Table S6.** Primers used in this study.

## Abbreviations

*S. cerevisiae*: *Saccharomyces cerevisiae*
RFP: red fluorescence protein
CHR: chromosome
TEL: telomere
CEN: centromere
