# Supplementary material for: Genome-wide landscape of position effects on heterogeneous gene expression in Saccharomyces cerevisiae
Source: Biotechnol Biofuels. 2017 Jul 18;10:189. doi: 10.1186/s13068-017-0872-3 (PMC5516366; doi:10.1186/s13068-017-0872-3)
Supplement: Supplementary file 1 — Additional file 1: Figure S1. Influence of the CYC1 terminator. Figure S2. Relative fluorescence intensities of the GFP and RFP at the same locus. Figure S3. The influence of marker gene on position effect. Table S1. Number of integrative loci of each chromosome. Table S2. The relative fluorescence intensity of 1044 loci. Table S3. Fit curve of the distribution of the numbers of positions with different relative fluorescence intensities. Table S4. The position effect of the loci with extreme low and extreme high expression. Table S5. The effect of different carbon sources on position effect of the loci in CHR II. Table S6. Primers used in this study. [file 13068_2017_872_MOESM1_ESM.docx]

**Supporting Information:**

**Genome-wide landscape of position effects on heterogeneous gene expression in *Saccharomyces cerevisiae***

Xiao-Le Wu^1,2^†, Bing-Zhi Li^1,2^†, Wen-Zheng Zhang^1,2^, Kai Song^1^, Hao Qi^1,2^, Junbiao Dai^3^, Ying-Jin Yuan^1,2^*

^1^Key Laboratory of Systems Bioengineering (Ministry of Education), School of Chemical Engineering and Technology, Tianjin University, Tianjin, 300072, PR China

^2^SynBio Research Platform, Collaborative Innovation Center of Chemical Science and Engineering (Tianjin), Tianjin University, Tianjin, 300072, PR China

^3^Key laboratory of Industrial Biocatalysis (Ministry of Education) and Center for Synthetic and Systems Biology, School of Life Sciences, Tsinghua University, Beijing 100084, PR China.

*Corresponding author: Y-J Yuan, E-mail: yjyuan@tju.edu.cn; Tel: 86-22-27403888

Fax: 86-22-27403389

†Equal contribution.

**CONTENTS OF ADDITIONAL INFORMATION**

Additional Tables

Table S1

Number of integrative loci of each chromosome

Table S2

The relative fluorescence intensity of 1044 loci

Table S3

Fit Curve of the distribution of the numbers of positions with different relative fluorescence intensities.

Table S4

The position effect of the loci with extreme low and extreme high expression

Table S5

The effect of different carbon sources on position effect of the loci in Chr *II*

Table S6

Primers used in this study

Additional Figures

Figure S1

Influence of the CYC1 terminator.

Figure S2

Relative fluorescence intensities of the GFP and RFP at the same locus.

Figure S3

The influence of marker gene on position effect.

Table S1. Number of integrative loci of each chromosome

| Chromosome | Size (kb)^a^ | Position^b^ | Chromosome | Size (kb) ^a^ | Position^b^ |
| --- | --- | --- | --- | --- | --- |
| Chr *I* | 230.2 | 19 | Chr *IX* | 439.8 | 39 |
| Chr *II* | 813.1 | 71 | Chr *X* | 745.7 | 63 |
| Chr *III* | 316.1 | 35 | Chr *XI* | 666.8 | 59 |
| Chr *IV* | 1531.9 | 131 | Chr *XII* | 1078.1 | 89 |
| Chr *V* | 576.8 | 50 | Chr *XIII* | 924.4 | 79 |
| Chr *VI* | 270.1 | 24 | Chr *XIV* | 784.3 | 69 |
| Chr *VII* | 1090.9 | 95 | Chr *XV* | 1091.2 | 93 |
| Chr *VIII* | 562.6 | 47 | Chr *XVI* | 948.0 | 81 |

^a^The size of each chromosome.

^b^The position number on each chromosome.

Table S2. The relative fluorescence intensity of 1044 loci

| **No.** | **ORF name** | Relative Fluorescence Intensit | |
| --- | --- | --- | --- |
|  |  | Average | Error |
| TH00001 | YAL068C | 4.77 | 0.36 |
| TH00002 | YAL065C | 5.02 | 0.20 |
| TH00003 | YAL062W | 5.85 | 1.19 |
| TH00004 | YAL060W | 6.28 | 0.16 |
| TH00005 | YAL053W | 5.81 | 0.23 |
| TH00006 | YAL044C | 8.49 | 0.12 |
| TH00007 | YAL037W | 6.39 | 0.23 |
| TH00008 | YAL031C | 7.29 | 0.24 |
| TH00009 | YAL026C | 6.83 | 0.36 |
| TH00010 | YAL023C | 6.76 | 0.39 |
| TH00011 | YAL018C | 5.93 | 0.21 |
| TH00012 | YAL011W | 6.14 | 0.09 |
| TH00013 | YAL002W | 6.27 | 0.42 |
| TH00014 | YAR002W | 7.06 | 0.61 |
| TH00015 | YAR003W | 7.37 | 0.69 |
| TH00016 | YAR014C | 7.32 | 0.53 |
| TH00017 | YAR023C | 5.91 | 0.69 |
| TH00018 | YAR042W | 8.48 | 0.03 |
| TH00019 | YAR050W | 5.61 | 0.29 |
| TH00020 | YBL107C | 5.06 | 0.49 |
| TH00021 | YBL106C | 5.90 | 0.15 |
| TH00022 | YBL102W | 5.92 | 0.10 |
| TH00023 | YBL099W | 8.88 | 0.53 |
| TH00024 | YBL091C | 6.22 | 0.22 |
| TH00025 | YBL087C | 4.47 | 0.61 |
| TH00026 | YBL081W | 5.04 | 0.21 |
| TH00027 | YBL075C | 6.30 | 0.38 |
| TH00028 | YBL067C | 6.10 | 0.14 |
| TH00029 | YBL060W | 7.04 | 1.03 |
| TH00030 | YBL052C | 4.98 | 0.53 |
| TH00031 | YBL046W | 5.52 | 0.61 |
| TH00032 | YBL039C | 3.22 | 0.12 |
| TH00033 | YBL036C | 6.65 | 0.36 |
| TH00034 | YBL028C | 6.61 | 0.13 |
| TH00035 | YBL022C | 7.14 | 0.06 |
| TH00036 | YBL016W | 6.43 | 0.08 |
| TH00037 | YBL011W | 7.27 | 0.29 |
| TH00038 | YBL005W | 8.52 | 0.33 |
| TH00039 | YBL003C | 6.28 | 0.31 |
| TH00040 | YBL001C | 5.62 | 0.15 |
| TH00041 | YBR001C | 1.54 | 0.27 |
| TH00042 | YBR008C | 7.23 | 0.51 |
| TH00043 | YBR013C | 6.28 | 0.73 |
| TH00044 | YBR018C | 6.38 | 0.21 |
| TH00045 | YBR024W | 5.57 | 0.55 |
| TH00046 | YBR033W | 6.26 | 0.69 |
| TH00047 | YBR037C | 6.89 | 0.47 |
| TH00048 | YBR044C | 7.11 | 0.32 |
| TH00049 | YBR050C | 5.89 | 0.56 |
| TH00050 | YBR056W | 5.64 | 0.61 |
| TH00051 | YBR059C | 5.28 | 0.51 |
| TH00052 | YBR067C | 6.14 | 0.36 |
| TH00053 | YBR073W | 7.51 | 0.37 |
| TH00054 | YBR078W | 6.56 | 0.44 |
| TH00055 | YBR083W | 6.10 | 0.23 |
| TH00056 | YBR085C-A | 5.90 | 0.42 |
| TH00057 | YBR093C | 6.12 | 0.12 |
| TH00058 | YBR098W | 7.01 | 0.29 |
| TH00059 | YBR108W | 5.67 | 0.57 |
| TH00060 | YBR114W | 5.69 | 0.32 |
| TH00061 | YBR120C | 6.53 | 0.56 |
| TH00062 | YBR128C | 12.92 | 0.18 |
| TH00063 | YBR131W | 6.63 | 0.15 |
| TH00064 | YBR139W | 6.22 | 0.29 |
| TH00065 | YBR141C | 6.81 | 0.10 |
| TH00066 | YBR149W | 6.69 | 0.58 |
| TH00067 | YBR156C | 6.96 | 0.11 |
| TH00068 | YBR158W | 6.24 | 0.60 |
| TH00069 | YBR162C | 5.74 | 0.08 |
| TH00070 | YBR169C | 6.00 | 0.16 |
| TH00071 | YBR180W | 6.04 | 0.41 |
| TH00072 | YBR185C | 5.80 | 0.09 |
| TH00073 | YBR195C | 6.92 | 0.08 |
| TH00074 | YBR201W | 6.38 | 0.27 |
| TH00075 | YBR207W | 5.11 | 0.49 |
| TH00076 | YBR212W | 6.21 | 0.34 |
| TH00077 | YBR218C | 6.09 | 0.12 |
| TH00078 | YBR225W | 6.30 | 0.02 |
| TH00079 | YBR233W | 5.56 | 0.03 |
| TH00080 | YBR238C | 5.50 | 0.04 |
| TH00081 | YBR244W | 5.97 | 0.72 |
| TH00082 | YBR250W | 6.86 | 0.33 |
| TH00083 | YBR259W | 7.84 | 1.05 |
| TH00084 | YBR270C | 8.14 | 0.48 |
| TH00085 | YBR275C | 7.23 | 0.34 |
| TH00086 | YBR282W | 4.29 | 0.19 |
| TH00087 | YBR289W | 7.70 | 0.55 |
| TH00088 | YBR294W | 5.20 | 0.26 |
| TH00089 | YBR298C | 4.96 | 0.76 |
| TH00090 | YBR301W | 4.61 | 0.12 |
| TH00091 | YCL076W | 3.76 | 0.36 |
| TH00092 | YCL074W | 4.27 | 0.00 |
| TH00093 | YCL069W | 3.91 | 0.04 |
| TH00094 | YCL058C | 5.53 | 0.29 |
| TH00095 | YCL051W | 6.42 | 0.16 |
| TH00096 | YCL044C | 6.54 | 0.26 |
| TH00097 | YCL036W | 5.72 | 0.50 |
| TH00098 | YCL027W | 7.38 | 0.42 |
| TH00099 | YCL025C | 5.99 | 0.16 |
| TH00100 | YCL021W-A | 7.66 | 0.42 |
| TH00101 | YCL016C | 7.05 | 0.37 |
| TH00102 | YCL005W | 5.74 | 0.47 |
| TH00103 | YCL001W-B | 5.29 | 0.07 |
| TH00104 | YCR001W | 5.67 | 0.32 |
| TH00105 | YCR004C | 6.41 | 0.22 |
| TH00106 | YCR010C | 6.82 | 0.51 |
| TH00107 | YCR011C | 9.68 | 0.20 |
| TH00108 | YCR016W | 8.17 | 0.31 |
| TH00109 | YCR021C | 6.76 | 0.30 |
| TH00110 | YCR024C-A | 5.01 | 0.30 |
| TH00111 | YCR027C | 5.58 | 0.26 |
| TH00112 | YCR031C | 4.41 | 0.00 |
| TH00113 | YCR032W | 5.69 | 0.73 |
| TH00114 | YCR034W | 6.57 | 0.16 |
| TH00115 | YCR043C | 6.89 | 0.56 |
| TH00116 | YCR051W | 8.14 | 0.12 |
| TH00117 | YCR061W | 5.08 | 0.32 |
| TH00118 | YCR071C | 9.18 | 0.49 |
| TH00119 | YCR077C | 5.14 | 0.30 |
| TH00120 | YCR087C-A | 6.10 | 0.41 |
| TH00121 | YCR091W | 5.79 | 0.08 |
| TH00122 | YCR095C | 5.07 | 0.29 |
| TH00123 | YCR099C | 5.06 | 0.21 |
| TH00124 | YCR106W | 5.73 | 0.52 |
| TH00125 | YCR107W | 5.07 | 0.59 |
| TH00126 | YDL243C | 5.50 | 0.60 |
| TH00127 | YDL240W | 5.96 | 0.41 |
| TH00128 | YDL234C | 6.29 | 0.00 |
| TH00129 | YDL227C | 6.38 | 0.27 |
| TH00130 | YDL223C | 7.11 | 0.24 |
| TH00131 | YDL215C | 5.83 | 0.57 |
| TH00132 | YDL210W | 5.79 | 0.24 |
| TH00133 | YDL204W | 6.18 | 0.36 |
| TH00134 | YDL194W | 5.35 | 0.56 |
| TH00135 | YDL190C | 6.36 | 0.82 |
| TH00136 | YDL183C | 6.69 | 0.24 |
| TH00137 | YDL176W | 8.88 | 0.02 |
| TH00138 | YDL170W | 5.15 | 0.04 |
| TH00139 | YDL161W | 5.90 | 0.45 |
| TH00140 | YDL154W | 5.85 | 0.05 |
| TH00141 | YDL146W | 5.57 | 0.01 |
| TH00142 | YDL142C | 8.12 | 0.19 |
| TH00143 | YDL137W | 5.98 | 0.13 |
| TH00144 | YDL131W | 1.23 | 0.27 |
| TH00145 | YDL124W | 5.95 | 0.44 |
| TH00146 | YDL116W | 5.76 | 0.05 |
| TH00147 | YDL110C | 6.46 | 0.52 |
| TH00148 | YDL104C | 6.94 | 0.08 |
| TH00149 | YDL095W | 5.42 | 0.49 |
| TH00150 | YDL088C | 5.64 | 0.19 |
| TH00151 | YDL080C | 6.52 | 0.29 |
| TH00152 | YDL074C | 9.40 | 0.43 |
| TH00153 | YDL065C | 6.52 | 0.07 |
| TH00154 | YDL059C | 6.48 | 0.06 |
| TH00155 | YDL054C | 1.55 | 0.10 |
| TH00156 | YDL045W-A | 8.51 | 0.35 |
| TH00157 | YDL039C | 6.97 | 0.39 |
| TH00158 | YDL033C | 6.14 | 0.04 |
| TH00159 | YDL024C | 6.31 | 0.68 |
| TH00160 | YDL019C | 6.46 | 0.04 |
| TH00161 | YDL010W | 6.26 | 0.02 |
| TH00162 | YDL002C | 5.92 | 0.76 |
| TH00163 | YDL001W | 5.95 | 0.40 |
| TH00164 | YDR001C | 5.74 | 0.05 |
| TH00165 | YDR004W | 6.76 | 0.26 |
| TH00166 | YDR010C | 7.26 | 0.33 |
| TH00167 | YDR011W | 8.38 | 0.47 |
| TH00168 | YDR017C | 6.52 | 0.66 |
| TH00169 | YDR025W | 5.70 | 0.06 |
| TH00170 | YDR031W | 6.48 | 0.42 |
| TH00171 | YDR032C | 6.43 | 0.67 |
| TH00172 | YDR034C | 4.91 | 0.09 |
| TH00173 | YDR036C | 6.34 | 0.58 |
| TH00174 | YDR042C | 6.23 | 0.65 |
| TH00175 | YDR049W | 6.16 | 0.24 |
| TH00176 | YDR055W | 6.21 | 0.04 |
| TH00177 | YDR061W | 6.05 | 0.01 |
| TH00178 | YDR070C | 6.16 | 0.20 |
| TH00179 | YDR076W | 6.20 | 0.56 |
| TH00180 | YDR083W | 5.16 | 0.01 |
| TH00181 | YDR089W | 6.44 | 0.46 |
| TH00182 | YDR096W | 5.98 | 0.15 |
| TH00183 | YDR098C | 7.08 | 0.58 |
| TH00184 | YDR104C | 7.32 | 0.69 |
| TH00185 | YDR108W | 6.44 | 0.25 |
| TH00186 | YDR117C | 9.08 | 0.02 |
| TH00187 | YDR122W | 6.13 | 0.46 |
| TH00188 | YDR128W | 6.01 | 0.26 |
| TH00189 | YDR132C | 6.33 | 0.62 |
| TH00190 | YDR139C | 6.05 | 0.32 |
| TH00191 | YDR144C | 5.96 | 0.02 |
| TH00192 | YDR148C | 6.23 | 0.59 |
| TH00193 | YDR155C | 5.75 | 0.22 |
| TH00194 | YDR161W | 6.48 | 0.37 |
| TH00195 | YDR169C | 5.90 | 0.43 |
| TH00196 | YDR171W | 6.08 | 0.32 |
| TH00197 | YDR176W | 5.87 | 0.22 |
| TH00198 | YDR182W-A | 5.90 | 0.04 |
| TH00199 | YDR191W | 4.91 | 0.34 |
| TH00200 | YDR198C | 6.68 | 0.31 |
| TH00201 | YDR206W | 8.05 | 0.66 |
| TH00202 | YDR210W | 5.45 | 0.58 |
| TH00203 | YDR213W | 5.41 | 0.38 |
| TH00204 | YDR217C | 6.36 | 0.06 |
| TH00205 | YDR223W | 6.70 | 0.42 |
| TH00206 | YDR229W | 6.48 | 0.02 |
| TH00207 | YDR237W | 7.51 | 0.01 |
| TH00208 | YDR242W | 6.21 | 0.34 |
| TH00209 | YDR249C | 5.21 | 0.32 |
| TH00210 | YDR257C | 6.19 | 0.12 |
| TH00211 | YDR261C | 6.26 | 0.34 |
| TH00212 | YDR264C | 6.50 | 0.40 |
| TH00213 | YDR270W | 6.27 | 0.07 |
| TH00214 | YDR279W | 5.73 | 0.65 |
| TH00215 | YDR284C | 5.33 | 0.43 |
| TH00216 | YDR291W | 6.35 | 0.56 |
| TH00217 | YDR296W | 6.67 | 0.41 |
| TH00218 | YDR304C | 6.01 | 0.06 |
| TH00219 | YDR309C | 6.69 | 0.54 |
| TH00220 | YDR314C | 7.58 | 0.49 |
| TH00221 | YDR318W | 6.22 | 0.38 |
| TH00222 | YDR323C | 8.66 | 0.34 |
| TH00223 | YDR330W | 5.66 | 0.22 |
| TH00224 | YDR334W | 8.23 | 0.19 |
| TH00225 | YDR338C | 5.90 | 0.03 |
| TH00226 | YDR345C | 5.48 | 0.48 |
| TH00227 | YDR349C | 5.39 | 0.29 |
| TH00228 | YDR357C | 7.23 | 0.31 |
| TH00229 | YDR363W | 2.84 | 0.29 |
| TH00230 | YDR368W | 7.19 | 0.31 |
| TH00231 | YDR374C | 5.71 | 0.27 |
| TH00232 | YDR380W | 5.90 | 0.07 |
| TH00233 | YDR387C | 5.70 | 0.07 |
| TH00234 | YDR393W | 6.19 | 0.32 |
| TH00235 | YDR400W | 5.87 | 0.34 |
| TH00236 | YDR406W | 5.23 | 0.12 |
| TH00237 | YDR414C | 6.57 | 0.49 |
| TH00238 | YDR420W | 6.02 | 0.54 |
| TH00239 | YDR424C | 5.16 | 0.35 |
| TH00240 | YDR435C | 6.71 | 0.39 |
| TH00241 | YDR441C | 5.01 | 0.24 |
| TH00242 | YDR448W | 11.18 | 1.23 |
| TH00243 | YDR456W | 5.60 | 0.32 |
| TH00244 | YDR458C | 6.11 | 0.47 |
| TH00245 | YDR465C | 6.37 | 0.36 |
| TH00246 | YDR471W | 6.79 | 0.10 |
| TH00247 | YDR479C | 6.89 | 0.05 |
| TH00248 | YDR486C | 5.58 | 0.24 |
| TH00249 | YDR495C | 8.18 | 0.04 |
| TH00250 | YDR501W | 6.24 | 0.39 |
| TH00251 | YDR507C | 6.12 | 0.29 |
| TH00252 | YDR516C | 6.30 | 0.09 |
| TH00253 | YDR524C | 6.62 | 0.26 |
| TH00254 | YDR533C | 7.40 | 0.32 |
| TH00255 | YDR539W | 6.06 | 0.19 |
| TH00256 | YDR541C | 6.07 | 0.20 |
| TH00257 | YEL072W | 6.43 | 0.10 |
| TH00258 | YEL067C | 5.77 | 0.40 |
| TH00259 | YEL066W | 5.93 | 0.35 |
| TH00260 | YEL062W | 6.31 | 0.33 |
| TH00261 | YEL056W | 6.49 | 0.33 |
| TH00262 | YEL050C | 6.62 | 0.46 |
| TH00263 | YEL043W | 6.02 | 0.01 |
| TH00264 | YEL036C | 6.81 | 0.49 |
| TH00265 | YEL030W | 6.89 | 0.38 |
| TH00266 | YEL024W | 6.55 | 0.36 |
| TH00267 | YEL020C | 6.77 | 0.57 |
| TH00268 | YEL012W | 7.02 | 0.51 |
| TH00269 | YEL006W | 6.05 | 0.60 |
| TH00270 | YEL001C | 5.42 | 0.37 |
| TH00271 | YER001W | 6.33 | 0.04 |
| TH00272 | YER007C-A | 6.65 | 0.43 |
| TH00273 | YER011W | 6.57 | 0.27 |
| TH00274 | YER019W | 7.57 | 0.01 |
| TH00275 | YER024W | 5.46 | 0.07 |
| TH00276 | YER031C | 6.05 | 0.17 |
| TH00277 | YER039C-A | 7.30 | 0.24 |
| TH00278 | YER045C | 6.17 | 0.59 |
| TH00279 | YER049W | 5.29 | 0.08 |
| TH00280 | YER054C | 6.35 | 0.41 |
| TH00281 | YER060W | 6.95 | 0.38 |
| TH00282 | YER065C | 7.07 | 0.08 |
| TH00283 | YER071C | 8.14 | 0.41 |
| TH00284 | YER076C | 6.53 | 0.06 |
| TH00285 | YER081W | 6.52 | 0.42 |
| TH00286 | YER089C | 6.98 | 0.02 |
| TH00287 | YER093C-A | 5.43 | 0.17 |
| TH00288 | YER099C | 6.24 | 0.17 |
| TH00289 | YER106W | 5.74 | 0.66 |
| TH00290 | YER111C | 6.91 | 0.30 |
| TH00291 | YER116C | 5.76 | 0.17 |
| TH00292 | YER124C | 6.51 | 0.60 |
| TH00293 | YER129W | 6.24 | 0.17 |
| TH00294 | YER132C | 8.02 | 0.07 |
| TH00295 | YER137C | 8.17 | 0.54 |
| TH00296 | YER142C | 5.88 | 0.20 |
| TH00297 | YER150W | 6.47 | 0.01 |
| TH00298 | YER155C | 5.36 | 0.50 |
| TH00299 | YER158C | 5.71 | 0.38 |
| TH00300 | YER163C | 6.44 | 0.59 |
| TH00301 | YER166W | 5.91 | 0.56 |
| TH00302 | YER170W | 6.50 | 0.35 |
| TH00303 | YER175C | 6.97 | 0.05 |
| TH00304 | YER182W | 6.32 | 0.65 |
| TH00305 | YER186C | 5.40 | 0.36 |
| TH00306 | YER188W | 5.10 | 0.20 |
| TH00307 | YFL056C | 5.27 | 0.31 |
| TH00308 | YFL053W | 5.78 | 0.25 |
| TH00309 | YFL050C | 5.79 | 0.27 |
| TH00310 | YFL042C | 6.33 | 0.56 |
| TH00311 | YFL036W | 8.70 | 0.34 |
| TH00312 | YFL033C | 6.87 | 0.06 |
| TH00313 | YFL026W | 4.76 | 0.23 |
| TH00314 | YFL021W | 6.24 | 0.03 |
| TH00315 | YFL013C | 5.72 | 0.30 |
| TH00316 | YFL007W | 5.57 | 0.10 |
| TH00317 | YFL004W | 6.03 | 0.49 |
| TH00318 | YFL001W | 5.99 | 0.42 |
| TH00319 | YFR001W | 4.43 | 0.40 |
| TH00320 | YFR006W | 5.27 | 0.36 |
| TH00321 | YFR012W | 5.57 | 0.32 |
| TH00322 | YFR016C | 5.51 | 0.11 |
| TH00323 | YFR021W | 6.58 | 0.45 |
| TH00324 | YFR025C | 5.77 | 0.23 |
| TH00325 | YFR030W | 5.77 | 0.34 |
| TH00326 | YFR036W | 6.13 | 0.28 |
| TH00327 | YFR043C | 5.21 | 0.42 |
| TH00328 | YFR053C | 5.25 | 0.07 |
| TH00329 | YFR055W | 4.65 | 0.37 |
| TH00330 | YFR057W | 4.75 | 0.10 |
| TH00331 | YGL263W | 4.04 | 0.13 |
| TH00332 | YGL258W | 5.40 | 0.56 |
| TH00333 | YGL253W | 6.75 | 0.44 |
| TH00334 | YGL248W | 5.66 | 0.03 |
| TH00335 | YGL241W | 7.17 | 0.36 |
| TH00336 | YGL232W | 6.23 | 0.01 |
| TH00337 | YGL227W | 6.37 | 0.39 |
| TH00338 | YGL219C | 5.57 | 0.08 |
| TH00339 | YGL209W | 5.23 | 0.25 |
| TH00340 | YGL205W | 6.76 | 0.29 |
| TH00341 | YGL202W | 6.25 | 0.02 |
| TH00342 | YGL196W | 6.18 | 0.63 |
| TH00343 | YGL192W | 5.54 | 0.55 |
| TH00344 | YGL184C | 6.88 | 0.46 |
| TH00345 | YGL178W | 6.64 | 0.32 |
| TH00346 | YGL170C | 6.81 | 0.35 |
| TH00347 | YGL164C | 6.08 | 0.35 |
| TH00348 | YGL159W | 6.21 | 0.53 |
| TH00349 | YGL154C | 5.66 | 0.36 |
| TH00350 | YGL147C | 5.30 | 0.30 |
| TH00351 | YGL141W | 5.60 | 0.82 |
| TH00352 | YGL138C | 5.63 | 0.04 |
| TH00353 | YGL131C | 6.51 | 0.66 |
| TH00354 | YGL124C | 6.03 | 0.71 |
| TH00355 | YGL117W | 5.58 | 0.08 |
| TH00356 | YGL110C | 6.04 | 0.28 |
| TH00357 | YGL101W | 6.84 | 0.33 |
| TH00358 | YGL096W | 5.85 | 0.27 |
| TH00359 | YGL094C | 8.72 | 0.20 |
| TH00360 | YGL086W | 6.75 | 0.41 |
| TH00361 | YGL079W | 6.30 | 0.22 |
| TH00362 | YGL066W | 7.08 | 0.33 |
| TH00363 | YGL063W | 6.60 | 0.68 |
| TH00364 | YGL056C | 6.46 | 0.29 |
| TH00365 | YGL049C | 5.62 | 0.08 |
| TH00366 | YGL043W | 6.45 | 0.40 |
| TH00367 | YGL035C | 2.88 | 0.47 |
| TH00368 | YGL027C | 5.69 | 0.08 |
| TH00369 | YGL021W | 6.79 | 0.35 |
| TH00370 | YGL014W | 5.60 | 0.40 |
| TH00371 | YGL007W | 6.01 | 0.31 |
| TH00372 | YGL004C | 5.19 | 0.67 |
| TH00373 | YGL002W | 5.89 | 0.11 |
| TH00374 | YGR001C | 5.45 | 0.07 |
| TH00375 | YGR004W | 6.07 | 0.01 |
| TH00376 | YGR012W | 6.81 | 0.50 |
| TH00377 | YGR021W | 7.12 | 0.04 |
| TH00378 | YGR027C | 5.72 | 0.15 |
| TH00379 | YGR032W | 5.74 | 0.31 |
| TH00380 | YGR038W | 1.11 | 0.28 |
| TH00381 | YGR040W | 6.34 | 0.41 |
| TH00382 | YGR049W | 5.89 | 0.31 |
| TH00383 | YGR055W | 5.80 | 0.26 |
| TH00384 | YGR061C | 9.01 | 0.01 |
| TH00385 | YGR067C | 4.63 | 0.23 |
| TH00386 | YGR072W | 8.91 | 0.45 |
| TH00387 | YGR084C | 7.39 | 0.02 |
| TH00388 | YGR087C | 5.76 | 0.28 |
| TH00389 | YGR089W | 5.19 | 0.01 |
| TH00390 | YGR093W | 6.53 | 0.09 |
| TH00391 | YGR097W | 6.67 | 0.18 |
| TH00392 | YGR104C | 8.38 | 0.92 |
| TH00393 | YGR109C | 6.88 | 0.75 |
| TH00394 | YGR112W | 6.01 | 0.09 |
| TH00395 | YGR121C | 7.18 | 0.41 |
| TH00396 | YGR125W | 5.99 | 0.34 |
| TH00397 | YGR132C | 6.52 | 0.25 |
| TH00398 | YGR141W | 5.63 | 0.01 |
| TH00399 | YGR144W | 5.72 | 0.21 |
| TH00400 | YGR149W | 5.84 | 0.21 |
| TH00401 | YGR157W | 9.63 | 0.11 |
| TH00402 | YGR161W-C | 6.32 | 0.09 |
| TH00403 | YGR163W | 6.32 | 0.30 |
| TH00404 | YGR170W | 6.83 | 0.56 |
| TH00405 | YGR178C | 6.46 | 0.43 |
| TH00406 | YGR184C | 9.57 | 0.33 |
| TH00407 | YGR189C | 6.21 | 0.22 |
| TH00408 | YGR194C | 6.46 | 0.34 |
| TH00409 | YGR200C | 6.70 | 0.00 |
| TH00410 | YGR209C | 6.73 | 0.30 |
| TH00411 | YGR217W | 5.92 | 0.03 |
| TH00412 | YGR220C | 5.83 | 0.10 |
| TH00413 | YGR227W | 6.20 | 0.18 |
| TH00414 | YGR234W | 6.14 | 0.12 |
| TH00415 | YGR240C | 12.23 | 1.76 |
| TH00416 | YGR247W | 6.50 | 0.01 |
| TH00417 | YGR250C | 1.48 | 0.57 |
| TH00418 | YGR258C | 5.15 | 0.32 |
| TH00419 | YGR263C | 5.49 | 0.18 |
| TH00420 | YGR271W | 5.48 | 0.24 |
| TH00421 | YGR276C | 6.66 | 0.52 |
| TH00422 | YGR281W | 5.62 | 0.07 |
| TH00423 | YGR287C | 4.88 | 0.14 |
| TH00424 | YGR292W | 4.80 | 0.17 |
| TH00425 | YGR295C | 6.16 | 0.07 |
| TH00426 | YHL047C | 4.96 | 0.49 |
| TH00427 | YHL046C | 5.63 | 0.28 |
| TH00428 | YHL039W | 6.93 | 0.34 |
| TH00429 | YHL033C | 4.49 | 0.52 |
| TH00430 | YHL028W | 4.99 | 0.44 |
| TH00431 | YHL023C | 6.16 | 0.35 |
| TH00432 | YHL017W | 6.00 | 0.49 |
| TH00433 | YHL009C | 4.82 | 0.03 |
| TH00434 | YHL006C | 6.06 | 0.15 |
| TH00435 | YHL001W | 6.33 | 0.50 |
| TH00436 | YHR001W | 6.02 | 0.39 |
| TH00437 | YHR001W-A | 5.38 | 0.54 |
| TH00438 | YHR007C-A | 6.25 | 0.43 |
| TH00439 | YHR014W | 6.14 | 0.28 |
| TH00440 | YHR018C | 5.90 | 0.21 |
| TH00441 | YHR022C-A | 5.62 | 0.37 |
| TH00442 | YHR029C | 6.80 | 0.45 |
| TH00443 | YHR035W | 5.88 | 0.35 |
| TH00444 | YHR043C | 5.82 | 0.17 |
| TH00445 | YHR048W | 4.39 | 0.16 |
| TH00446 | YHR057C | 5.93 | 0.20 |
| TH00447 | YHR064C | 3.84 | 0.43 |
| TH00448 | YHR073W | 6.19 | 0.22 |
| TH00449 | YHR076W | 6.44 | 0.32 |
| TH00450 | YHR080C | 6.75 | 0.49 |
| TH00451 | YHR086W | 6.11 | 0.73 |
| TH00452 | YHR092C | 6.83 | 0.31 |
| TH00453 | YHR098C | 5.76 | 0.08 |
| TH00454 | YHR100C | 8.06 | 0.52 |
| TH00455 | YHR104W | 7.27 | 0.27 |
| TH00456 | YHR112C | 5.93 | 0.18 |
| TH00457 | YHR117W | 6.11 | 0.34 |
| TH00458 | YHR126C | 6.49 | 0.37 |
| TH00459 | YHR134W | 6.19 | 0.12 |
| TH00460 | YHR142W | 10.10 | 0.00 |
| TH00461 | YHR149C | 5.72 | 0.03 |
| TH00462 | YHR155W | 6.43 | 0.20 |
| TH00463 | YHR160C | 6.32 | 0.02 |
| TH00464 | YHR167W | 6.08 | 0.19 |
| TH00465 | YHR171W | 7.27 | 0.46 |
| TH00466 | YHR178W | 9.17 | 0.29 |
| TH00467 | YHR181W | 6.86 | 0.53 |
| TH00468 | YHR187W | 6.76 | 0.57 |
| TH00469 | YHR195W | 6.44 | 0.03 |
| TH00470 | YHR203C | 5.55 | 0.12 |
| TH00471 | YHR207C | 6.09 | 0.57 |
| TH00472 | YHR210C | 7.83 | 0.21 |
| TH00473 | YIL173W | 6.96 | 0.01 |
| TH00474 | YIL170W | 4.87 | 0.06 |
| TH00475 | YIL164C | 6.60 | 0.26 |
| TH00476 | YIL156W | 6.48 | 0.39 |
| TH00477 | YIL152W | 6.98 | 0.19 |
| TH00478 | YIL148W | 8.23 | 0.00 |
| TH00479 | YIL140W | 5.85 | 0.03 |
| TH00480 | YIL135C | 6.10 | 0.52 |
| TH00481 | YIL130W | 6.51 | 0.01 |
| TH00482 | YIL127C | 5.85 | 0.12 |
| TH00483 | YIL122W | 5.39 | 0.16 |
| TH00484 | YIL116W | 8.35 | 0.16 |
| TH00485 | YIL110W | 5.12 | 0.14 |
| TH00486 | YIL105C | 6.89 | 0.01 |
| TH00487 | YIL098C | 7.00 | 0.63 |
| TH00488 | YIL092W | 1.19 | 0.07 |
| TH00489 | YIL084C | 5.85 | 0.64 |
| TH00490 | YIL077C | 5.84 | 0.56 |
| TH00491 | YIL072W | 5.66 | 0.06 |
| TH00492 | YIL066C | 5.69 | 0.13 |
| TH00493 | YIL055C | 8.23 | 0.32 |
| TH00494 | YIL047C | 6.18 | 0.62 |
| TH00495 | YIL042C | 5.98 | 0.14 |
| TH00496 | YIL035C | 6.42 | 0.29 |
| TH00497 | YIL029C | 5.59 | 0.53 |
| TH00498 | YIL023C | 6.38 | 0.25 |
| TH00499 | YIL015W | 5.97 | 0.67 |
| TH00500 | YIL009C-A | 8.47 | 0.09 |
| TH00501 | YIL005W | 5.47 | 0.11 |
| TH00502 | YIL001W | 5.88 | 0.27 |
| TH00503 | YIR001C | 6.29 | 0.39 |
| TH00504 | YIR002C | 5.31 | 0.47 |
| TH00505 | YIR007W | 6.67 | 0.49 |
| TH00506 | YIR017C | 6.40 | 0.26 |
| TH00507 | YIR021W | 6.33 | 0.01 |
| TH00508 | YIR027C | 5.71 | 0.36 |
| TH00509 | YIR034C | 5.80 | 0.30 |
| TH00510 | YIR039C | 6.40 | 0.66 |
| TH00511 | YIR044C | 5.20 | 0.41 |
| TH00512 | YJL218W | 5.95 | 0.41 |
| TH00513 | YJL212C | 5.00 | 0.47 |
| TH00514 | YJL207C | 6.66 | 0.12 |
| TH00515 | YJL198W | 6.35 | 0.49 |
| TH00516 | YJL193W | 5.86 | 0.10 |
| TH00517 | YJL183W | 7.80 | 0.21 |
| TH00518 | YJL176C | 8.11 | 0.38 |
| TH00519 | YJL165C | 6.02 | 0.69 |
| TH00520 | YJL159W | 6.59 | 0.07 |
| TH00521 | YJL154C | 9.13 | 0.81 |
| TH00522 | YJL146W | 8.15 | 0.26 |
| TH00523 | YJL141C | 7.07 | 0.40 |
| TH00524 | YJL137C | 6.28 | 0.38 |
| TH00525 | YJL131C | 7.40 | 0.12 |
| TH00526 | YJL128C | 5.61 | 0.32 |
| TH00527 | YJL118W | 4.79 | 0.17 |
| TH00528 | YJL112W | 7.14 | 0.27 |
| TH00529 | YJL108C | 6.82 | 0.41 |
| TH00530 | YJL103C | 6.40 | 0.39 |
| TH00531 | YJL099W | 6.89 | 0.55 |
| TH00532 | YJL094C | 5.62 | 0.04 |
| TH00533 | YJL089W | 5.26 | 0.37 |
| TH00534 | YJL083W | 6.32 | 0.01 |
| TH00535 | YJL078C | 6.11 | 0.21 |
| TH00536 | YJL071W | 6.21 | 0.34 |
| TH00537 | YJL068C | 6.76 | 0.24 |
| TH00538 | YJL060W | 7.72 | 0.45 |
| TH00539 | YJL053W | 9.04 | 0.56 |
| TH00540 | YJL048C | 6.04 | 0.90 |
| TH00541 | YJL043W | 6.54 | 0.09 |
| TH00542 | YJL038C | 7.30 | 0.07 |
| TH00543 | YJL030W | 6.66 | 0.19 |
| TH00544 | YJL023C | 5.92 | 0.58 |
| TH00545 | YJL016W | 6.74 | 0.74 |
| TH00546 | YJL012C | 2.73 | 0.01 |
| TH00547 | YJL004C | 5.57 | 0.41 |
| TH00548 | YJL003W | 5.55 | 0.68 |
| TH00549 | YJR001W | 5.79 | 0.28 |
| TH00550 | YJR004C | 6.24 | 0.34 |
| TH00551 | YJR010W | 7.79 | 0.42 |
| TH00552 | YJR019C | 6.55 | 0.45 |
| TH00553 | YJR030C | 5.30 | 0.18 |
| TH00554 | YJR032W | 5.52 | 0.08 |
| TH00555 | YJR036C | 5.06 | 0.17 |
| TH00556 | YJR043C | 8.12 | 0.70 |
| TH00557 | YJR049C | 6.44 | 0.18 |
| TH00558 | YJR055W | 8.67 | 0.40 |
| TH00559 | YJR061W | 6.85 | 0.51 |
| TH00560 | YJR066W | 6.66 | 0.30 |
| TH00561 | YJR077C | 7.48 | 0.32 |
| TH00562 | YJR088C | 7.25 | 0.40 |
| TH00563 | YJR092W | 6.00 | 0.03 |
| TH00564 | YJR095W | 7.41 | 0.26 |
| TH00565 | YJR105W | 7.73 | 0.27 |
| TH00566 | YJR111C | 5.41 | 0.59 |
| TH00567 | YJR120W | 5.49 | 0.14 |
| TH00568 | YJR127C | 6.05 | 0.04 |
| TH00569 | YJR133W | 6.04 | 0.59 |
| TH00570 | YJR137C | 6.96 | 0.73 |
| TH00571 | YJR142W | 5.87 | 0.04 |
| TH00572 | YJR149W | 5.71 | 0.03 |
| TH00573 | YJR152W | 5.56 | 0.52 |
| TH00574 | YJR154W | 5.06 | 0.13 |
| TH00575 | YKL222C | 5.27 | 0.01 |
| TH00576 | YKL220C | 5.88 | 0.27 |
| TH00577 | YKL217W | 5.44 | 0.71 |
| TH00578 | YKL212W | 6.47 | 0.17 |
| TH00579 | YKL208W | 7.09 | 0.17 |
| TH00580 | YKL204W | 7.03 | 0.04 |
| TH00581 | YKL197C | 6.03 | 0.19 |
| TH00582 | YKL190W | 7.44 | 0.46 |
| TH00583 | YKL185W | 5.42 | 0.21 |
| TH00584 | YKL183W | 6.19 | 0.05 |
| TH00585 | YKL175W | 2.32 | 0.01 |
| TH00586 | YKL168C | 6.26 | 0.15 |
| TH00587 | YKL162C | 6.23 | 0.43 |
| TH00588 | YKL157W | 6.19 | 0.57 |
| TH00589 | YKL149C | 6.60 | 0.51 |
| TH00590 | YKL142W | 5.67 | 0.36 |
| TH00591 | YKL133C | 6.17 | 0.46 |
| TH00592 | YKL127W | 6.49 | 0.64 |
| TH00593 | YKL121W | 6.56 | 0.33 |
| TH00594 | YKL110C | 5.97 | 0.06 |
| TH00595 | YKL105C | 5.91 | 0.26 |
| TH00596 | YKL101W | 6.25 | 0.55 |
| TH00597 | YKL094W | 6.63 | 0.30 |
| TH00598 | YKL087C | 8.66 | 0.08 |
| TH00599 | YKL077W | 6.48 | 0.60 |
| TH00600 | YKL072W | 6.21 | 0.28 |
| TH00601 | YKL068W-A | 5.96 | 0.15 |
| TH00602 | YKL062W | 5.32 | 0.60 |
| TH00603 | YKL055C | 4.36 | 0.38 |
| TH00604 | YKL048C | 5.80 | 0.66 |
| TH00605 | YKL041W | 6.07 | 0.65 |
| TH00606 | YKL034W | 5.68 | 0.11 |
| TH00607 | YKL029C | 5.99 | 0.43 |
| TH00608 | YKL023W | 7.21 | 0.30 |
| TH00609 | YKL017C | 6.26 | 0.02 |
| TH00610 | YKL010C | 6.77 | 0.14 |
| TH00611 | YKL006W | 4.33 | 0.15 |
| TH00612 | YKL001C | 6.09 | 0.20 |
| TH00613 | YKR001C | 5.06 | 0.21 |
| TH00614 | YKR003W | 5.76 | 0.50 |
| TH00615 | YKR009C | 5.84 | 0.73 |
| TH00616 | YKR015C | 6.20 | 0.02 |
| TH00617 | YKR021W | 6.72 | 0.31 |
| TH00618 | YKR027W | 6.29 | 0.05 |
| TH00619 | YKR031C | 6.48 | 0.06 |
| TH00620 | YKR039W | 6.50 | 0.32 |
| TH00621 | YKR050W | 4.69 | 0.25 |
| TH00622 | YKR054C | 6.82 | 0.26 |
| TH00623 | YKR057W | 4.38 | 0.13 |
| TH00624 | YKR064W | 5.98 | 0.63 |
| TH00625 | YKR072C | 5.18 | 0.54 |
| TH00626 | YKR078W | 5.84 | 0.22 |
| TH00627 | YKR085C | 5.75 | 0.51 |
| TH00628 | YKR091W | 6.10 | 0.08 |
| TH00629 | YKR095W | 5.61 | 0.38 |
| TH00630 | YKR099W | 4.85 | 0.05 |
| TH00631 | YKR102W | 4.56 | 0.10 |
| TH00632 | YKR105C | 4.72 | 0.00 |
| TH00633 | YKR106W | 5.63 | 0.14 |
| TH00634 | YLL063C | 5.13 | 0.54 |
| TH00635 | YLL058W | 5.33 | 0.67 |
| TH00636 | YLL052C | 5.34 | 0.01 |
| TH00637 | YLL045C | 4.81 | 0.29 |
| TH00638 | YLL040C | 4.89 | 0.34 |
| TH00639 | YLL032C | 5.26 | 0.15 |
| TH00640 | YLL028W | 5.24 | 0.61 |
| TH00641 | YLL024C | 5.70 | 0.39 |
| TH00642 | YLL019C | 6.51 | 0.02 |
| TH00643 | YLL014W | 5.18 | 0.41 |
| TH00644 | YLL010C | 5.23 | 0.35 |
| TH00645 | YLL002W | 5.98 | 0.27 |
| TH00646 | YLL001W | 5.97 | 0.06 |
| TH00647 | YLR001C | 5.17 | 0.07 |
| TH00648 | YLR003C | 6.73 | 0.11 |
| TH00649 | YLR011W | 5.83 | 0.66 |
| TH00650 | YLR019W | 5.45 | 0.26 |
| TH00651 | YLR024C | 4.43 | 0.16 |
| TH00652 | YLR030W | 6.07 | 0.28 |
| TH00653 | YLR035C | 6.01 | 0.07 |
| TH00654 | YLR039C | 8.59 | 0.59 |
| TH00655 | YLR046C | 5.80 | 0.06 |
| TH00656 | YLR055C | 6.75 | 0.43 |
| TH00657 | YLR064W | 5.98 | 0.58 |
| TH00658 | YLR072W | 6.15 | 0.05 |
| TH00659 | YLR080W | 5.14 | 0.13 |
| TH00660 | YLR085C | 5.47 | 0.45 |
| TH00661 | YLR089C | 4.94 | 0.35 |
| TH00662 | YLR092W | 6.60 | 0.16 |
| TH00663 | YLR097C | 7.47 | 0.45 |
| TH00664 | YLR104W | 6.92 | 0.45 |
| TH00665 | YLR107W | 8.29 | 0.36 |
| TH00666 | YLR113W | 7.79 | 0.66 |
| TH00667 | YLR118C | 6.95 | 0.67 |
| TH00668 | YLR125W | 6.32 | 0.04 |
| TH00669 | YLR133W | 8.26 | 0.55 |
| TH00670 | YLR138W | 5.76 | 0.60 |
| TH00671 | YLR146C | 6.28 | 0.62 |
| TH00672 | YLR152C | 5.81 | 0.09 |
| TH00673 | YLR164W | 6.70 | 0.01 |
| TH00674 | YLR173W | 6.75 | 0.76 |
| TH00675 | YLR180W | 8.51 | 0.21 |
| TH00676 | YLR188W | 6.36 | 0.68 |
| TH00677 | YLR193C | 7.16 | 0.48 |
| TH00678 | YLR204W | 6.32 | 0.47 |
| TH00679 | YLR211C | 7.37 | 0.67 |
| TH00680 | YLR219W | 6.89 | 0.83 |
| TH00681 | YLR225C | 7.16 | 0.07 |
| TH00682 | YLR231C | 6.38 | 0.25 |
| TH00683 | YLR237W | 6.72 | 0.52 |
| TH00684 | YLR241W | 5.74 | 0.01 |
| TH00685 | YLR248W | 5.98 | 0.60 |
| TH00686 | YLR254C | 5.80 | 0.10 |
| TH00687 | YLR257W | 6.19 | 0.18 |
| TH00688 | YLR263W | 6.49 | 0.41 |
| TH00689 | YLR271W | 6.30 | 0.21 |
| TH00690 | YLR278C | 4.88 | 0.29 |
| TH00691 | YLR285W | 6.94 | 0.71 |
| TH00692 | YLR292C | 7.35 | 0.58 |
| TH00693 | YLR303W | 6.38 | 0.67 |
| TH00694 | YLR306W | 6.53 | 0.00 |
| TH00695 | YLR312C | 5.84 | 0.68 |
| TH00696 | YLR313C | 5.13 | 0.28 |
| TH00697 | YLR318W | 5.23 | 0.44 |
| TH00698 | YLR324W | 7.65 | 0.24 |
| TH00699 | YLR332W | 6.56 | 0.10 |
| TH00700 | YLR341W | 6.56 | 0.64 |
| TH00701 | YLR342W-A | 6.31 | 0.58 |
| TH00702 | YLR350W | 6.55 | 0.35 |
| TH00703 | YLR356W | 8.01 | 0.64 |
| TH00704 | YLR363C | 6.49 | 0.58 |
| TH00705 | YLR371W | 0.98 | 0.02 |
| TH00706 | YLR380W | 7.01 | 0.14 |
| TH00707 | YLR384C | 6.05 | 0.38 |
| TH00708 | YLR389C | 6.67 | 0.45 |
| TH00709 | YLR395C | 7.22 | 0.12 |
| TH00710 | YLR401C | 8.12 | 0.65 |
| TH00711 | YLR408C | 5.88 | 0.11 |
| TH00712 | YLR412W | 5.76 | 0.59 |
| TH00713 | YLR419W | 6.50 | 0.42 |
| TH00714 | YLR422W | 6.66 | 0.02 |
| TH00715 | YLR425W | 7.64 | 0.30 |
| TH00716 | YLR429W | 6.19 | 0.06 |
| TH00717 | YLR437C | 6.25 | 0.11 |
| TH00718 | YLR441C | 4.56 | 0.25 |
| TH00719 | YLR449W | 6.22 | 0.09 |
| TH00720 | YLR453C | 5.73 | 0.68 |
| TH00721 | YLR456W | 6.72 | 0.21 |
| TH00722 | YLR461W | 5.24 | 0.51 |
| TH00723 | YML131W | 5.82 | 0.50 |
| TH00724 | YML123C | 6.21 | 0.19 |
| TH00725 | YML117W | 7.08 | 0.37 |
| TH00726 | YML111W | 6.74 | 0.03 |
| TH00727 | YML104C | 5.75 | 0.12 |
| TH00728 | YML100W | 5.55 | 0.11 |
| TH00729 | YML094W | 5.49 | 0.04 |
| TH00730 | YML086C | 5.70 | 0.07 |
| TH00731 | YML081W | 7.06 | 0.14 |
| TH00732 | YML074C | 7.28 | 0.19 |
| TH00733 | YML070W | 7.20 | 0.45 |
| TH00734 | YML067C | 4.50 | 0.52 |
| TH00735 | YML059C | 11.39 | 0.20 |
| TH00736 | YML054C-A | 5.93 | 0.17 |
| TH00737 | YML047C | 6.25 | 0.20 |
| TH00738 | YML042W | 6.76 | 0.09 |
| TH00739 | YML038C | 6.50 | 0.09 |
| TH00740 | YML030W | 7.20 | 0.64 |
| TH00741 | YML022W | 6.11 | 0.20 |
| TH00742 | YML016C | 7.42 | 0.07 |
| TH00743 | YML008C | 8.23 | 0.06 |
| TH00744 | YML003W | 5.73 | 0.39 |
| TH00745 | YML002W | 5.80 | 0.32 |
| TH00746 | YMR001C-A | 6.56 | 0.53 |
| TH00747 | YMR006C | 6.54 | 0.16 |
| TH00748 | YMR011W | 6.96 | 0.02 |
| TH00749 | YMR016C | 7.81 | 0.39 |
| TH00750 | YMR018W | 5.47 | 0.16 |
| TH00751 | YMR025W | 6.40 | 0.64 |
| TH00752 | YMR032W | 5.68 | 0.13 |
| TH00753 | YMR038C | 9.16 | 0.33 |
| TH00754 | YMR044W | 7.37 | 0.47 |
| TH00755 | YMR048W | 6.71 | 0.07 |
| TH00756 | YMR054W | 5.60 | 0.54 |
| TH00757 | YMR063W | 7.68 | 0.34 |
| TH00758 | YMR069W | 5.90 | 0.01 |
| TH00759 | YMR077C | 5.39 | 0.33 |
| TH00760 | YMR081C | 5.68 | 0.37 |
| TH00761 | YMR088C | 6.97 | 0.38 |
| TH00762 | YMR095C | 2.22 | 0.02 |
| TH00763 | YMR101C | 8.79 | 0.19 |
| TH00764 | YMR106C | 6.44 | 0.45 |
| TH00765 | YMR110C | 5.98 | 0.76 |
| TH00766 | YMR118C | 6.85 | 0.30 |
| TH00767 | YMR124W | 5.59 | 0.70 |
| TH00768 | YMR129W | 8.06 | 0.26 |
| TH00769 | YMR135C | 7.57 | 0.33 |
| TH00770 | YMR143W | 5.83 | 0.08 |
| TH00771 | YMR153W | 8.75 | 0.00 |
| TH00772 | YMR160W | 6.16 | 0.44 |
| TH00773 | YMR164C | 6.55 | 0.56 |
| TH00774 | YMR169C | 7.57 | 0.24 |
| TH00775 | YMR175W-A | 6.51 | 0.24 |
| TH00776 | YMR181C | 4.13 | 0.01 |
| TH00777 | YMR188C | 5.94 | 0.24 |
| TH00778 | YMR192W | 7.74 | 0.14 |
| TH00779 | YMR198W | 7.93 | 0.55 |
| TH00780 | YMR206W | 7.85 | 0.45 |
| TH00781 | YMR209C | 7.44 | 0.18 |
| TH00782 | YMR214W | 6.96 | 0.40 |
| TH00783 | YMR219W | 6.67 | 0.19 |
| TH00784 | YMR225C | 6.85 | 0.45 |
| TH00785 | YMR230W | 7.87 | 0.32 |
| TH00786 | YMR237W | 7.27 | 0.00 |
| TH00787 | YMR243C | 7.44 | 0.14 |
| TH00788 | YMR247W-A | 6.51 | 0.04 |
| TH00789 | YMR255W | 6.62 | 0.17 |
| TH00790 | YMR261C | 7.34 | 0.46 |
| TH00791 | YMR271C | 6.61 | 0.49 |
| TH00792 | YMR274C | 7.18 | 0.71 |
| TH00793 | YMR280C | 5.46 | 0.32 |
| TH00794 | YMR285C | 7.36 | 0.00 |
| TH00795 | YMR291W | 7.26 | 0.33 |
| TH00796 | YMR299C | 8.53 | 0.40 |
| TH00797 | YMR305C | 6.79 | 0.20 |
| TH00798 | YMR307W | 9.49 | 0.18 |
| TH00799 | YMR313C | 6.79 | 0.28 |
| TH00800 | YMR318C | 6.50 | 0.59 |
| TH00801 | YMR322C | 6.77 | 0.46 |
| TH00802 | YNL339C | 5.48 | 0.15 |
| TH00803 | YNL334C | 6.51 | 0.39 |
| TH00804 | YNL328C | 6.92 | 0.15 |
| TH00805 | YNL321W | 7.23 | 0.19 |
| TH00806 | YNL311C | 9.18 | 0.28 |
| TH00807 | YNL304W | 8.55 | 0.49 |
| TH00808 | YNL297C | 7.10 | 0.64 |
| TH00809 | YNL291C | 5.84 | 0.09 |
| TH00810 | YNL286W | 6.91 | 0.61 |
| TH00811 | YNL281W | 6.54 | 0.12 |
| TH00812 | YNL275W | 7.33 | 0.09 |
| TH00813 | YNL271C | 7.60 | 0.59 |
| TH00814 | YNL265C | 5.22 | 0.24 |
| TH00815 | YNL259C | 6.31 | 0.05 |
| TH00816 | YNL254C | 7.57 | 0.68 |
| TH00817 | YNL249C | 5.79 | 0.12 |
| TH00818 | YNL241C | 5.74 | 0.51 |
| TH00819 | YNL233W | 6.98 | 0.67 |
| TH00820 | YNL229C | 5.81 | 0.44 |
| TH00821 | YNL224C | 5.66 | 0.28 |
| TH00822 | YNL217W | 6.44 | 0.54 |
| TH00823 | YNL209W | 5.86 | 0.36 |
| TH00824 | YNL200C | 6.26 | 0.01 |
| TH00825 | YNL193W | 6.01 | 0.23 |
| TH00826 | YNL187W | 6.34 | 0.20 |
| TH00827 | YNL180C | 8.02 | 0.13 |
| TH00828 | YNL173C | 6.48 | 0.41 |
| TH00829 | YNL167C | 7.17 | 0.05 |
| TH00830 | YNL160W | 8.27 | 0.40 |
| TH00831 | YNL148C | 6.43 | 0.16 |
| TH00832 | YNL141W | 5.52 | 0.14 |
| TH00833 | YNL135C | 6.31 | 0.19 |
| TH00834 | YNL127W | 6.16 | 0.70 |
| TH00835 | YNL123W | 6.92 | 0.32 |
| TH00836 | YNL116W | 6.78 | 0.01 |
| TH00837 | YNL107W | 6.10 | 0.67 |
| TH00838 | YNL101W | 6.08 | 0.03 |
| TH00839 | YNL096C | 4.35 | 0.67 |
| TH00840 | YNL090W | 6.92 | 0.31 |
| TH00841 | YNL085W | 6.48 | 0.05 |
| TH00842 | YNL078W | 5.52 | 0.62 |
| TH00843 | YNL071W | 5.87 | 0.07 |
| TH00844 | YNL065W | 4.82 | 0.30 |
| TH00845 | YNL058C | 8.27 | 0.55 |
| TH00846 | YNL054W | 7.14 | 0.73 |
| TH00847 | YNL047C | 7.32 | 0.39 |
| TH00848 | YNL041C | 6.73 | 0.25 |
| TH00849 | YNL035C | 5.79 | 0.26 |
| TH00850 | YNL031C | 5.55 | 0.01 |
| TH00851 | YNL024C | 6.20 | 0.03 |
| TH00852 | YNL016W | 5.32 | 0.50 |
| TH00853 | YNL011C | 5.64 | 0.81 |
| TH00854 | YNL004W | 6.87 | 0.25 |
| TH00855 | YNL001W | 4.03 | 0.35 |
| TH00856 | YNR001C | 4.81 | 0.49 |
| TH00857 | YNR004W | 7.11 | 0.75 |
| TH00858 | YNR009W | 6.07 | 0.33 |
| TH00859 | YNR012W | 6.16 | 0.11 |
| TH00860 | YNR018W | 6.72 | 0.24 |
| TH00861 | YNR024W | 7.13 | 0.47 |
| TH00862 | YNR031C | 4.34 | 0.35 |
| TH00863 | YNR037C | 9.19 | 0.33 |
| TH00864 | YNR045W | 8.95 | 0.71 |
| TH00865 | YNR052C | 6.25 | 0.22 |
| TH00866 | YNR056C | 8.40 | 0.22 |
| TH00867 | YNR062C | 6.43 | 0.13 |
| TH00868 | YNR067C | 6.49 | 0.15 |
| TH00869 | YNR070W | 5.28 | 0.15 |
| TH00870 | YNR075W | 4.62 | 0.51 |
| TH00871 | YOL164W-A | 5.63 | 0.46 |
| TH00872 | YOL162W | 6.40 | 0.58 |
| TH00873 | YOL158C | 5.54 | 0.30 |
| TH00874 | YOL154W | 4.96 | 0.09 |
| TH00875 | YOL147C | 6.65 | 0.37 |
| TH00876 | YOL140W | 7.04 | 0.24 |
| TH00877 | YOL132W | 6.13 | 0.35 |
| TH00878 | YOL124C | 5.56 | 0.19 |
| TH00879 | YOL119C | 8.38 | 0.43 |
| TH00880 | YOL111C | 7.59 | 0.20 |
| TH00881 | YOL104C | 7.23 | 0.35 |
| TH00882 | YOL100W | 6.60 | 0.43 |
| TH00883 | YOL092W | 6.88 | 0.57 |
| TH00884 | YOL087C | 8.81 | 0.51 |
| TH00885 | YOL082W | 6.79 | 0.37 |
| TH00886 | YOL080C | 6.24 | 0.20 |
| TH00887 | YOL073C | 6.16 | 0.09 |
| TH00888 | YOL067C | 7.41 | 0.02 |
| TH00889 | YOL060C | 8.15 | 0.16 |
| TH00890 | YOL054W | 5.56 | 0.15 |
| TH00891 | YOL048C | 7.24 | 0.57 |
| TH00892 | YOL041C | 4.28 | 0.26 |
| TH00893 | YOL031C | 5.76 | 0.36 |
| TH00894 | YOL025W | 5.99 | 0.00 |
| TH00895 | YOL019W-A | 6.23 | 0.43 |
| TH00896 | YOL014W | 5.38 | 0.52 |
| TH00897 | YOL007C | 5.79 | 0.25 |
| TH00898 | YOL002C | 5.76 | 0.52 |
| TH00899 | YOL001W | 5.87 | 0.80 |
| TH00900 | YOR001W | 5.56 | 0.37 |
| TH00901 | YOR005C | 5.56 | 0.05 |
| TH00902 | YOR010C | 4.45 | 0.72 |
| TH00903 | YOR016C | 6.73 | 0.12 |
| TH00904 | YOR020W-A | 6.45 | 0.21 |
| TH00905 | YOR022C | 5.24 | 0.27 |
| TH00906 | YOR028C | 5.33 | 0.24 |
| TH00907 | YOR034C | 6.40 | 0.29 |
| TH00908 | YOR040W | 5.73 | 0.04 |
| TH00909 | YOR049C | 6.03 | 0.16 |
| TH00910 | YOR058C | 5.91 | 0.57 |
| TH00911 | YOR062C | 6.19 | 0.47 |
| TH00912 | YOR070C | 5.74 | 0.41 |
| TH00913 | YOR076C | 6.40 | 0.34 |
| TH00914 | YOR083W | 6.40 | 0.30 |
| TH00915 | YOR090C | 7.55 | 0.17 |
| TH00916 | YOR094W | 6.31 | 0.17 |
| TH00917 | YOR101W | 7.66 | 0.26 |
| TH00918 | YOR109W | 7.24 | 0.15 |
| TH00919 | YOR115C | 6.63 | 0.75 |
| TH00920 | YOR120W | 6.38 | 0.63 |
| TH00921 | YOR128C | 5.66 | 0.18 |
| TH00922 | YOR133W | 6.14 | 0.17 |
| TH00923 | YOR140W | 5.60 | 0.10 |
| TH00924 | YOR144C | 7.75 | 0.61 |
| TH00925 | YOR150W | 7.20 | 0.43 |
| TH00926 | YOR154W | 6.28 | 0.60 |
| TH00927 | YOR161C | 7.17 | 0.56 |
| TH00928 | YOR166C | 6.03 | 0.26 |
| TH00929 | YOR175C | 7.06 | 0.12 |
| TH00930 | YOR179C | 4.38 | 0.40 |
| TH00931 | YOR187W | 5.93 | 0.07 |
| TH00932 | YOR191W | 4.82 | 0.52 |
| TH00933 | YOR193W | 5.20 | 0.52 |
| TH00934 | YOR201C | 6.10 | 0.01 |
| TH00935 | YOR208W | 5.60 | 0.59 |
| TH00936 | YOR213C | 5.39 | 0.06 |
| TH00937 | YOR220W | 5.92 | 0.13 |
| TH00938 | YOR228C | 4.44 | 0.49 |
| TH00939 | YOR234C | 6.15 | 0.26 |
| TH00940 | YOR243C | 5.49 | 0.38 |
| TH00941 | YOR252W | 6.20 | 0.14 |
| TH00942 | YOR264W | 6.08 | 0.12 |
| TH00943 | YOR269W | 6.84 | 0.63 |
| TH00944 | YOR274W | 7.43 | 0.40 |
| TH00945 | YOR288C | 8.53 | 0.58 |
| TH00946 | YOR291W | 7.04 | 0.17 |
| TH00947 | YOR298W | 7.85 | 0.52 |
| TH00948 | YOR304C-A | 7.89 | 0.53 |
| TH00949 | YOR312C | 5.44 | 0.49 |
| TH00950 | YOR317W | 9.11 | 0.56 |
| TH00951 | YOR324C | 8.24 | 0.08 |
| TH00952 | YOR328W | 6.68 | 0.23 |
| TH00953 | YOR334W | 8.00 | 0.38 |
| TH00954 | YOR339C | 7.99 | 0.67 |
| TH00955 | YOR344C | 7.98 | 0.44 |
| TH00956 | YOR347C | 8.19 | 0.42 |
| TH00957 | YOR351C | 7.97 | 0.32 |
| TH00958 | YOR356W | 7.97 | 0.44 |
| TH00959 | YOR363C | 8.50 | 0.52 |
| TH00960 | YOR371C | 7.91 | 0.25 |
| TH00961 | YOR376W-A | 6.97 | 0.47 |
| TH00962 | YOR381W | 7.67 | 0.50 |
| TH00963 | YOR386W | 6.53 | 0.49 |
| TH00964 | YPL277C | 5.89 | 0.00 |
| TH00965 | YPL274W | 8.64 | 0.52 |
| TH00966 | YPL269W | 7.80 | 0.55 |
| TH00967 | YPL262W | 8.50 | 0.12 |
| TH00968 | YPL257W | 7.86 | 0.05 |
| TH00969 | YPL253C | 7.80 | 0.39 |
| TH00970 | YPL247C | 7.74 | 0.06 |
| TH00971 | YPL241C | 1.10 | 0.23 |
| TH00972 | YPL232W | 7.98 | 0.19 |
| TH00973 | YPL227C | 7.69 | 0.49 |
| TH00974 | YPL222W | 8.66 | 0.65 |
| TH00975 | YPL216W | 6.58 | 0.13 |
| TH00976 | YPL208W | 7.46 | 0.30 |
| TH00977 | YPL202C | 6.28 | 0.34 |
| TH00978 | YPL194W | 8.40 | 0.15 |
| TH00979 | YPL187W | 6.27 | 0.41 |
| TH00980 | YPL181W | 5.81 | 0.23 |
| TH00981 | YPL176C | 7.55 | 0.42 |
| TH00982 | YPL170W | 7.88 | 0.52 |
| TH00983 | YPL164C | 7.09 | 0.11 |
| TH00984 | YPL159C | 7.96 | 0.25 |
| TH00985 | YPL152W-A | 6.89 | 0.53 |
| TH00986 | YPL145C | 7.85 | 0.42 |
| TH00987 | YPL140C | 6.65 | 0.64 |
| TH00988 | YPL133C | 6.40 | 0.01 |
| TH00989 | YPL127C | 5.77 | 0.03 |
| TH00990 | YPL119C-A | 7.27 | 0.52 |
| TH00991 | YPL113C | 7.79 | 0.21 |
| TH00992 | YPL108W | 8.16 | 0.27 |
| TH00993 | YPL101W | 7.99 | 0.32 |
| TH00994 | YPL092W | 6.74 | 0.08 |
| TH00995 | YPL087W | 8.03 | 0.23 |
| TH00996 | YPL084W | 7.05 | 0.42 |
| TH00997 | YPL078C | 9.29 | 0.04 |
| TH00998 | YPL071C | 6.67 | 0.55 |
| TH00999 | YPL061W | 5.86 | 0.23 |
| TH01000 | YPL059W | 7.13 | 0.16 |
| TH01001 | YPL054W | 7.08 | 0.43 |
| TH01002 | YPL045W | 9.26 | 0.25 |
| TH01003 | YPL038W-A | 6.58 | 0.65 |
| TH01004 | YPL031C | 7.54 | 0.49 |
| TH01005 | YPL024W | 7.68 | 0.50 |
| TH01006 | YPL019C | 7.66 | 0.11 |
| TH01007 | YPL014W | 11.14 | 0.08 |
| TH01008 | YPL008W | 7.65 | 0.35 |
| TH01009 | YPL003W | 7.78 | 0.28 |
| TH01010 | YPL001W | 7.05 | 0.45 |
| TH01011 | YPR001W | 5.59 | 0.13 |
| TH01012 | YPR004C | 6.30 | 0.02 |
| TH01013 | YPR009W | 7.34 | 0.46 |
| TH01014 | YPR015C | 6.29 | 0.17 |
| TH01015 | YPR020W | 5.71 | 0.07 |
| TH01016 | YPR024W | 7.66 | 0.17 |
| TH01017 | YPR028W | 10.09 | 0.04 |
| TH01018 | YPR032W | 6.97 | 0.75 |
| TH01019 | YPR040W | 9.17 | 0.11 |
| TH01020 | YPR047W | 7.67 | 0.41 |
| TH01021 | YPR057W | 9.02 | 0.49 |
| TH01022 | YPR068C | 7.98 | 0.47 |
| TH01023 | YPR075C | 7.86 | 0.58 |
| TH01024 | YPR084W | 7.44 | 0.20 |
| TH01025 | YPR093C | 5.37 | 0.15 |
| TH01026 | YPR101W | 7.01 | 0.01 |
| TH01027 | YPR108W-A | 8.93 | 0.25 |
| TH01028 | YPR115W | 6.31 | 0.26 |
| TH01029 | YPR118W | 7.49 | 0.69 |
| TH01030 | YPR121W | 7.64 | 0.03 |
| TH01031 | YPR128C | 7.63 | 0.61 |
| TH01032 | YPR135W | 7.62 | 0.61 |
| TH01033 | YPR140W | 8.84 | 0.27 |
| TH01034 | YPR148C | 2.75 | 0.28 |
| TH01035 | YPR156C | 7.97 | 0.36 |
| TH01036 | YPR159W | 8.60 | 0.16 |
| TH01037 | YPR160W | 7.14 | 0.86 |
| TH01038 | YPR166C | 2.33 | 0.17 |
| TH01039 | YPR174C | 7.54 | 0.29 |
| TH01040 | YPR184W | 7.14 | 0.07 |
| TH01041 | YPR188C | 7.86 | 0.76 |
| TH01042 | YPR193C | 7.07 | 0.39 |
| TH01043 | YPR198W | 7.54 | 0.39 |
| TH01044 | YPR201W | 6.29 | 0.33 |

Table S3. Fit Curve of the distribution of the numbers of positions within different relative fluorescence intensities.

|  | Fit Curve |
| --- | --- |
| Model | Gauss |
| Equation | y=y0 + (A/(w*sqrt(PI/2)))*exp(-2*((x-xc)/w)^2) |
| Reduced Chi-Sqr | 251.156 |
| Adj. R-Square | 0.98594 |

Table S4. The position effect of the loci with extreme low and extreme high expression

| **No.** | **ORF name** | Relative Fluorescence Intensit | |
| --- | --- | --- | --- |
|  |  | **Average** | **Error** |
| 1 | YAL068C | 4.77 | 0.36 |
| 2 | YAR042W | 8.48 | 0.03 |
| 3 | YBL099W | 8.88 | 0.53 |
| 4 | YBL087C | 4.47 | 0.61 |
| 5 | YBL052C | 4.98 | 0.53 |
| 6 | YBL039C | 3.22 | 0.12 |
| 7 | YBL005W | 8.52 | 0.33 |
| 8 | YBR001C | 1.54 | 0.27 |
| 9 | YBR128C | 12.92 | 0.18 |
| 10 | YBR270C | 8.14 | 0.48 |
| 11 | YBR282W | 4.29 | 0.19 |
| 12 | YBR298C | 4.96 | 0.76 |
| 13 | YBR301W | 4.61 | 0.12 |
| 14 | YCL076W | 3.76 | 0.36 |
| 15 | YCL074W | 4.27 | 0.00 |
| 16 | YCL069W | 3.91 | 0.04 |
| 17 | YCR011C | 9.68 | 0.20 |
| 18 | YCR031C | 4.41 | 0.00 |
| 19 | YCR051W | 8.14 | 0.12 |
| 20 | YCR071C | 9.18 | 0.49 |
| 21 | YDL142C | 8.12 | 0.19 |
| 22 | YDL131W | 1.23 | 0.27 |
| 23 | YDL074C | 9.40 | 0.43 |
| 24 | YDL054C | 1.55 | 0.10 |
| 25 | YDL045W-A | 8.51 | 0.35 |
| 26 | YDR011W | 8.38 | 0.47 |
| 27 | YDR034C | 4.91 | 0.09 |
| 28 | YDR117C | 9.08 | 0.02 |
| 29 | YDR191W | 4.91 | 0.34 |
| 30 | YDR323C | 8.66 | 0.34 |
| 31 | YDR334W | 8.23 | 0.19 |
| 32 | YDR363W | 2.84 | 0.29 |
| 33 | YDR448W | 11.18 | 1.23 |
| 34 | YDR495C | 8.18 | 0.04 |
| 35 | YER132C | 8.02 | 0.07 |
| 36 | YFL026W | 4.76 | 0.23 |
| 37 | YFR001W | 4.43 | 0.40 |
| 38 | YFR055W | 4.65 | 0.37 |
| 39 | YFR057W | 4.75 | 0.10 |
| 40 | YGL263W | 4.04 | 0.13 |
| 41 | YGL035C | 2.88 | 0.47 |
| 42 | YGR038W | 1.11 | 0.28 |
| 43 | YGR061C | 9.01 | 0.01 |
| 44 | YGR067C | 4.63 | 0.23 |
| 45 | YGR072W | 8.91 | 0.45 |
| 46 | YGR104C | 8.38 | 0.92 |
| 47 | YGR157W | 9.63 | 0.11 |
| 48 | YGR240C | 12.23 | 1.76 |
| 49 | YGR250C | 1.48 | 0.57 |
| 50 | YGR287C | 4.88 | 0.14 |
| 51 | YGR292W | 4.80 | 0.17 |
| 52 | YHL047C | 4.96 | 0.49 |
| 53 | YHL033C | 4.49 | 0.52 |
| 54 | YHL028W | 4.99 | 0.44 |
| 55 | YHL009C | 4.82 | 0.03 |
| 56 | YHR048W | 4.39 | 0.16 |
| 57 | YHR064C | 3.84 | 0.43 |
| 58 | YHR100C | 8.06 | 0.52 |
| 59 | YHR142W | 10.10 | 0.00 |
| 60 | YHR178W | 9.17 | 0.29 |
| 61 | YIL170W | 4.87 | 0.06 |
| 62 | YIL092W | 1.19 | 0.07 |
| 63 | YIL055C | 8.23 | 0.32 |
| 64 | YIL009C-A | 8.47 | 0.09 |
| 65 | YJL176C | 8.11 | 0.38 |
| 66 | YJL154C | 9.13 | 0.81 |
| 67 | YJL118W | 4.79 | 0.17 |
| 68 | YJL053W | 9.04 | 0.56 |
| 69 | YJL012C | 2.73 | 0.01 |
| 70 | YKL175W | 2.32 | 0.01 |
| 71 | YKL055C | 4.36 | 0.38 |
| 72 | YKL006W | 4.33 | 0.15 |
| 73 | YKR050W | 4.69 | 0.25 |
| 74 | YKR057W | 4.38 | 0.13 |
| 75 | YKR099W | 4.85 | 0.05 |
| 76 | YKR102W | 4.56 | 0.10 |
| 77 | YKR105C | 4.72 | 0.00 |
| 78 | YLL045C | 4.81 | 0.29 |
| 79 | YLL040C | 4.89 | 0.34 |
| 80 | YLR024C | 4.43 | 0.16 |
| 81 | YLR089C | 4.94 | 0.35 |
| 82 | YLR107W | 8.29 | 0.36 |
| 83 | YLR180W | 8.51 | 0.21 |
| 84 | YLR278C | 4.88 | 0.29 |
| 85 | YLR356W | 8.01 | 0.64 |
| 86 | YLR371W | 0.98 | 0.06 |
| 87 | YLR401C | 8.12 | 0.65 |
| 88 | YLR441C | 4.56 | 0.25 |
| 89 | YML067C | 4.50 | 0.52 |
| 90 | YML059C | 11.39 | 0.20 |
| 91 | YML008C | 8.23 | 0.06 |
| 92 | YMR038C | 9.16 | 0.33 |
| 93 | YMR095C | 2.22 | 0.02 |
| 94 | YMR101C | 8.79 | 0.19 |
| 95 | YMR129W | 8.06 | 0.26 |
| 96 | YMR181C | 4.13 | 0.01 |
| 97 | YMR299C | 8.53 | 0.40 |
| 98 | YMR307W | 9.49 | 0.18 |
| 99 | YNL304W | 8.55 | 0.49 |
| 100 | YNL180C | 8.02 | 0.13 |
| 101 | YNL160W | 8.27 | 0.40 |
| 102 | YNL096C | 4.35 | 0.67 |
| 103 | YNL065W | 4.82 | 0.30 |
| 104 | YNL058C | 8.27 | 0.55 |
| 105 | YNL001W | 4.03 | 0.35 |
| 106 | YNR001C | 4.81 | 0.49 |
| 107 | YNR031C | 4.34 | 0.35 |
| 108 | YNR037C | 9.19 | 0.33 |
| 109 | YNR045W | 8.95 | 0.71 |
| 110 | YNR056C | 8.40 | 0.22 |
| 111 | YNR075W | 4.62 | 0.51 |
| 112 | YOL154W | 4.96 | 0.09 |
| 113 | YOL119C | 8.38 | 0.43 |
| 114 | YOL087C | 8.81 | 0.51 |
| 115 | YOL060C | 8.15 | 0.16 |
| 116 | YOL041C | 4.28 | 0.26 |
| 117 | YOR010C | 4.45 | 0.72 |
| 118 | YOR179C | 4.38 | 0.40 |
| 119 | YOR191W | 4.82 | 0.52 |
| 120 | YOR228C | 4.44 | 0.49 |
| 121 | YOR288C | 8.53 | 0.58 |
| 122 | YOR317W | 9.11 | 0.56 |
| 123 | YOR324C | 8.24 | 0.08 |
| 124 | YOR334W | 8.17 | 0.54 |
| 125 | YOR339C | 8.12 | 0.70 |
| 126 | YOR344C | 8.35 | 0.16 |
| 127 | YOR347C | 8.19 | 0.42 |
| 128 | YOR351C | 8.15 | 0.26 |
| 129 | YOR356W | 8.67 | 0.40 |
| 130 | YOR363C | 8.50 | 0.52 |
| 131 | YOR371C | 8.75 | 0.00 |
| 132 | YPL274W | 8.64 | 0.52 |
| 133 | YPL262W | 8.50 | 0.12 |
| 134 | YPL257W | 8.17 | 0.31 |
| 135 | YPL253C | 9.57 | 0.33 |
| 136 | YPL247C | 8.49 | 0.12 |
| 137 | YPL241C | 1.10 | 0.23 |
| 138 | YPL227C | 8.26 | 0.55 |
| 139 | YPL222W | 8.66 | 0.65 |
| 140 | YPL194W | 8.40 | 0.15 |
| 141 | YPL108W | 8.16 | 0.27 |
| 142 | YPL087W | 8.03 | 0.23 |
| 143 | YPL078C | 9.29 | 0.04 |
| 144 | YPL045W | 9.26 | 0.25 |
| 145 | YPL024W | 9.18 | 0.28 |
| 146 | YPL019C | 8.72 | 0.20 |
| 147 | YPL014W | 11.14 | 0.08 |
| 148 | YPR024W | 8.59 | 0.59 |
| 149 | YPR028W | 10.09 | 0.04 |
| 150 | YPR040W | 9.17 | 0.11 |
| 151 | YPR057W | 9.02 | 0.49 |
| 152 | YPR108W-A | 8.93 | 0.25 |
| 153 | YPR121W | 8.14 | 0.41 |
| 154 | YPR128C | 8.05 | 0.66 |
| 155 | YPR135W | 8.88 | 0.02 |
| 156 | YPR140W | 8.84 | 0.27 |
| 157 | YPR148C | 2.75 | 0.28 |
| 158 | YPR159W | 8.60 | 0.16 |
| 159 | YPR160W | 8.70 | 0.34 |
| 160 | YPR166C | 2.33 | 0.17 |
| 161 | YPR184W | 8.23 | 0.00 |
| 162 | YPR193C | 8.66 | 0.08 |

Table S5. The effect of different carbon sources on position effect of the loci in Chr *II*

| No. | Gene | SD-LEU | | YPG | | RATIO |
| --- | --- | --- | --- | --- | --- | --- |
|  |  | Average | Error | Average | Error |  |
| TH00020 | YBL107C | 5.06 | 0.49 | 5.63 | 0.33 | 0.90 |
| TH00021 | YBL106C | 5.90 | 0.15 | 5.89 | 0.59 | 1.00 |
| TH00022 | YBL102W | 5.92 | 0.10 | 7.64 | 0.91 | 0.78 |
| TH00023 | YBL099W | 8.88 | 0.53 | 6.93 | 0.86 | 1.28 |
| TH00024 | YBL091C | 6.22 | 0.22 | 5.44 | 0.67 | 1.14 |
| TH00025 | YBL087C | 4.47 | 0.61 | 5.06 | 0.85 | 0.88 |
| TH00026 | YBL081W | 5.04 | 0.21 | 4.70 | 0.25 | 1.07 |
| TH00027 | YBL075C | 6.30 | 0.38 | 5.80 | 0.63 | 1.09 |
| TH00028 | YBL067C | 6.10 | 0.14 | 7.00 | 0.86 | 0.87 |
| TH00029 | YBL060W | 7.04 | 1.03 | 8.52 | 0.53 | 0.83 |
| TH00030 | YBL052C | 4.98 | 0.53 | 5.05 | 0.84 | 0.99 |
| TH00031 | YBL046W | 5.52 | 0.61 | 8.73 | 0.50 | 0.63 |
| TH00032 | YBL039C | 3.22 | 0.12 | 4.41 | 0.15 | 0.73 |
| TH00033 | YBL036C | 6.65 | 0.36 | 5.32 | 0.77 | 1.25 |
| TH00034 | YBL028C | 6.61 | 0.13 | 4.94 | 0.26 | 1.34 |
| TH00035 | YBL022C | 7.14 | 0.06 | 5.79 | 0.44 | 1.23 |
| TH00036 | YBL016W | 6.43 | 0.08 | 6.56 | 0.17 | 0.98 |
| TH00037 | YBL011W | 7.27 | 0.29 | 4.89 | 0.71 | 1.49 |
| TH00038 | YBL005W | 8.52 | 0.33 | 5.00 | 0.18 | 1.71 |
| TH00039 | YBL003C | 6.28 | 0.31 | 4.04 | 0.36 | 1.56 |
| TH00040 | YBL001C | 5.62 | 0.15 | 5.80 | 0.10 | 0.97 |
| TH00041 | YBR001C | 1.54 | 0.27 | 2.56 | 0.20 | 0.60 |
| TH00042 | YBR008C | 7.23 | 0.51 | 4.67 | 0.43 | 1.55 |
| TH00043 | YBR013C | 6.28 | 0.73 | 5.03 | 0.96 | 1.25 |
| TH00044 | YBR018C | 6.38 | 0.21 | 4.42 | 0.56 | 1.44 |
| TH00045 | YBR024W | 5.57 | 0.55 | 4.56 | 0.22 | 1.22 |
| TH00046 | YBR033W | 6.26 | 0.69 | 4.42 | 0.39 | 1.42 |
| TH00047 | YBR037C | 6.89 | 0.47 | 5.27 | 0.09 | 1.31 |
| TH00048 | YBR044C | 7.11 | 0.32 | 4.23 | 0.17 | 1.68 |
| TH00049 | YBR050C | 5.89 | 0.56 | 6.07 | 0.48 | 0.97 |
| TH00050 | YBR056W | 5.64 | 0.61 | 4.17 | 0.44 | 1.35 |
| TH00051 | YBR059C | 5.28 | 0.51 | 4.34 | 0.61 | 1.22 |
| TH00052 | YBR067C | 6.14 | 0.36 | 6.62 | 0.55 | 0.93 |
| TH00053 | YBR073W | 7.51 | 0.37 | 5.37 | 0.57 | 1.40 |
| TH00054 | YBR078W | 6.56 | 0.44 | 8.03 | 0.45 | 0.82 |
| TH00055 | YBR083W | 6.10 | 0.23 | 4.56 | 0.44 | 1.34 |
| TH00056 | YBR085C-A | 5.90 | 0.42 | 4.77 | 0.54 | 1.24 |
| TH00057 | YBR093C | 6.12 | 0.12 | 4.38 | 0.37 | 1.40 |
| TH00058 | YBR098W | 7.01 | 0.29 | 4.80 | 0.56 | 1.46 |
| TH00059 | YBR108W | 5.67 | 0.57 | 4.01 | 0.73 | 1.41 |
| TH00060 | YBR114W | 5.69 | 0.32 | 4.10 | 0.59 | 1.39 |
| TH00061 | YBR120C | 6.53 | 0.56 | 4.53 | 0.40 | 1.44 |
| TH00062 | YBR128C | 12.92 | 0.18 | 13.62 | 0.59 | 0.95 |
| TH00063 | YBR131W | 6.63 | 0.15 | 8.43 | 0.49 | 0.79 |
| TH00064 | YBR139W | 6.22 | 0.29 | 5.26 | 0.08 | 1.18 |
| TH00065 | YBR141C | 6.81 | 0.10 | 4.66 | 0.79 | 1.46 |
| TH00066 | YBR149W | 6.69 | 0.58 | 5.83 | 0.88 | 1.15 |
| TH00067 | YBR156C | 6.96 | 0.11 | 9.04 | 0.67 | 0.77 |
| TH00068 | YBR158W | 6.24 | 0.60 | 7.62 | 0.05 | 0.82 |
| TH00069 | YBR162C | 5.74 | 0.08 | 5.37 | 0.21 | 1.07 |
| TH00070 | YBR169C | 6.00 | 0.16 | 4.60 | 0.50 | 1.30 |
| TH00071 | YBR180W | 6.04 | 0.41 | 4.13 | 0.36 | 1.46 |
| TH00072 | YBR185C | 5.80 | 0.09 | 4.47 | 0.71 | 1.30 |
| TH00073 | YBR195C | 6.92 | 0.08 | 4.70 | 0.16 | 1.47 |
| TH00074 | YBR201W | 6.38 | 0.27 | 4.63 | 0.51 | 1.38 |
| TH00075 | YBR207W | 5.11 | 0.49 | 5.96 | 0.37 | 0.86 |
| TH00076 | YBR212W | 6.21 | 0.34 | 5.24 | 0.52 | 1.18 |
| TH00077 | YBR218C | 6.09 | 0.12 | 5.36 | 0.38 | 1.14 |
| TH00078 | YBR225W | 6.30 | 0.02 | 5.00 | 0.37 | 1.26 |
| TH00079 | YBR233W | 5.56 | 0.03 | 4.36 | 0.57 | 1.28 |
| TH00080 | YBR238C | 5.50 | 0.04 | 4.59 | 0.11 | 1.20 |
| TH00081 | YBR244W | 5.97 | 0.72 | 4.95 | 0.32 | 1.21 |
| TH00082 | YBR250W | 6.86 | 0.33 | 7.99 | 0.63 | 0.86 |
| TH00083 | YBR259W | 7.84 | 1.05 | 9.21 | 0.53 | 0.85 |
| TH00084 | YBR270C | 8.14 | 0.48 | 5.17 | 0.20 | 1.57 |
| TH00085 | YBR275C | 7.23 | 0.34 | 4.80 | 0.39 | 1.51 |
| TH00086 | YBR282W | 4.29 | 0.19 | 3.72 | 0.13 | 1.15 |
| TH00087 | YBR289W | 7.70 | 0.55 | 6.50 | 0.18 | 1.19 |
| TH00088 | YBR294W | 5.20 | 0.26 | 4.30 | 0.66 | 1.21 |
| TH00089 | YBR298C | 4.96 | 0.76 | 3.98 | 0.31 | 1.24 |
| TH00090 | YBR301W | 4.61 | 0.12 | 4.20 | 0.42 | 1.10 |

Table S6**.** Primers used in this study

|  | Primer sequences |
| --- | --- |
| YZ-URA3P-592-F | TCGGTAATCTCCGAACAGAAGGAAG |
| YZ-RFP-592-R | TGCATAACTGGACCATCTGATGGA |
| YZ-KANMX4-560-F | AAACGCTCCCCTCACAGACG |
| YZ-KANMX4-560-R | CAAACAGGAATCGAATGCAACCG |





Figure S1. Influence of the CYC1 terminator.


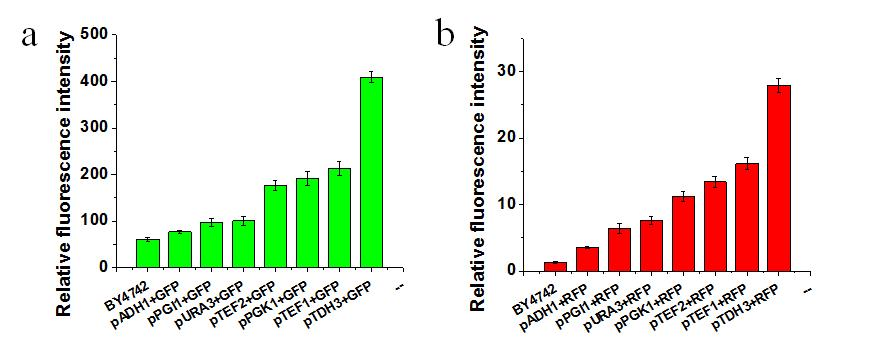


Figure S2**.** Relative fluorescence intensities of the GFP (a) and RFP (b) at the same locus.





Figure S3**.** The influence of marker gene on position effect.

Report gene was integrated with *LEU2* (red).

Report gene was integrated without *LEU2* (blue).
